# Unlocking the potential of soil microbial communities for bioremediation of emerging organic contaminants: omics-based approaches

**DOI:** 10.1186/s12934-024-02485-z

**Published:** 2024-07-25

**Authors:** Fatemeh Alidoosti, Minoo Giyahchi, Shabnam Moien, Hamid Moghimi

**Affiliations:** Department of Microbiology, School of Biology, College of Science, University of Tehran, Tehran, Iran

**Keywords:** Bioremediation, Emerging contaminants, Omics, Soil microbial communities

## Abstract

The remediation of emerging contaminants presents a pressing environmental challenge, necessitating innovative approaches for effective mitigation. This review article delves into the untapped potential of soil microbial communities in the bioremediation of emerging contaminants. Bioremediation, while a promising method, often proves time-consuming and requires a deep comprehension of microbial intricacies for enhancement. Given the challenges presented by the inability to culture many of these microorganisms, conventional methods are inadequate for achieving this goal. While omics-based methods provide an innovative approach to understanding the fundamental aspects, processes, and connections among microorganisms that are essential for improving bioremediation strategies. By exploring the latest advancements in omics technologies, this review aims to shed light on how these approaches can unlock the hidden capabilities of soil microbial communities, paving the way for more efficient and sustainable remediation solutions.

## Introduction

“Emerging contaminants (ECs)” are pollutants that have a new origin, alternate route to humans, or require new techniques for treatment [1]. These pollutants are divided into different categories according to their chemical properties and sources, including organic, inorganic, biological emerging contaminants, and other unknown compositions like micro and nanoplastics. Emerging inorganic contaminants include engineered nanoparticles, radionuclides, and nuclear wastes. Biological contaminants such as pathogenic bacteria, antibiotic-resistant bacteria and resistance genes, viruses, and protein contaminants [2]. Emerging organic contaminants (EOCs) encompass a wide range of chemical compounds, including pharmaceuticals and personal care products (PPCPs), pesticides, endocrine disrupting compounds (EDCs), surfactants, flame retardants, plasticizers, and industrial additives, among others. Metabolites and intermediate degradation products of the original compounds are also part of the EOCs [3]. They can be produced and released from households, hospitals, laboratory wastewater, agricultural processes, construction, landscaping transportation, or the food industry [1, 4–6]. Due to its unique filtering and buffering characteristics, the soil can absorb and retain most anthropogenic substances causing their accumulation in inland areas [7].

There are various health risks associated with EOCs, both in the short-term and long-term. These risks include abnormal physiological processes, higher cancer rates, increased toxicity potential of chemical mixtures, endocrine-disrupting effects, birth defects, infant malformations, and diabetes [1, 8]. Moreover, frequent exposure to antibiotics as an emerging pollutant can lead to antibiotic resistance in specific pathogenic microorganisms, making their treatment challenging [7, 8]. Considering their hazardous effects on humans and other biota, finding a remedial solution for removing EOCs sounds urgent.

**Fig. 1:**
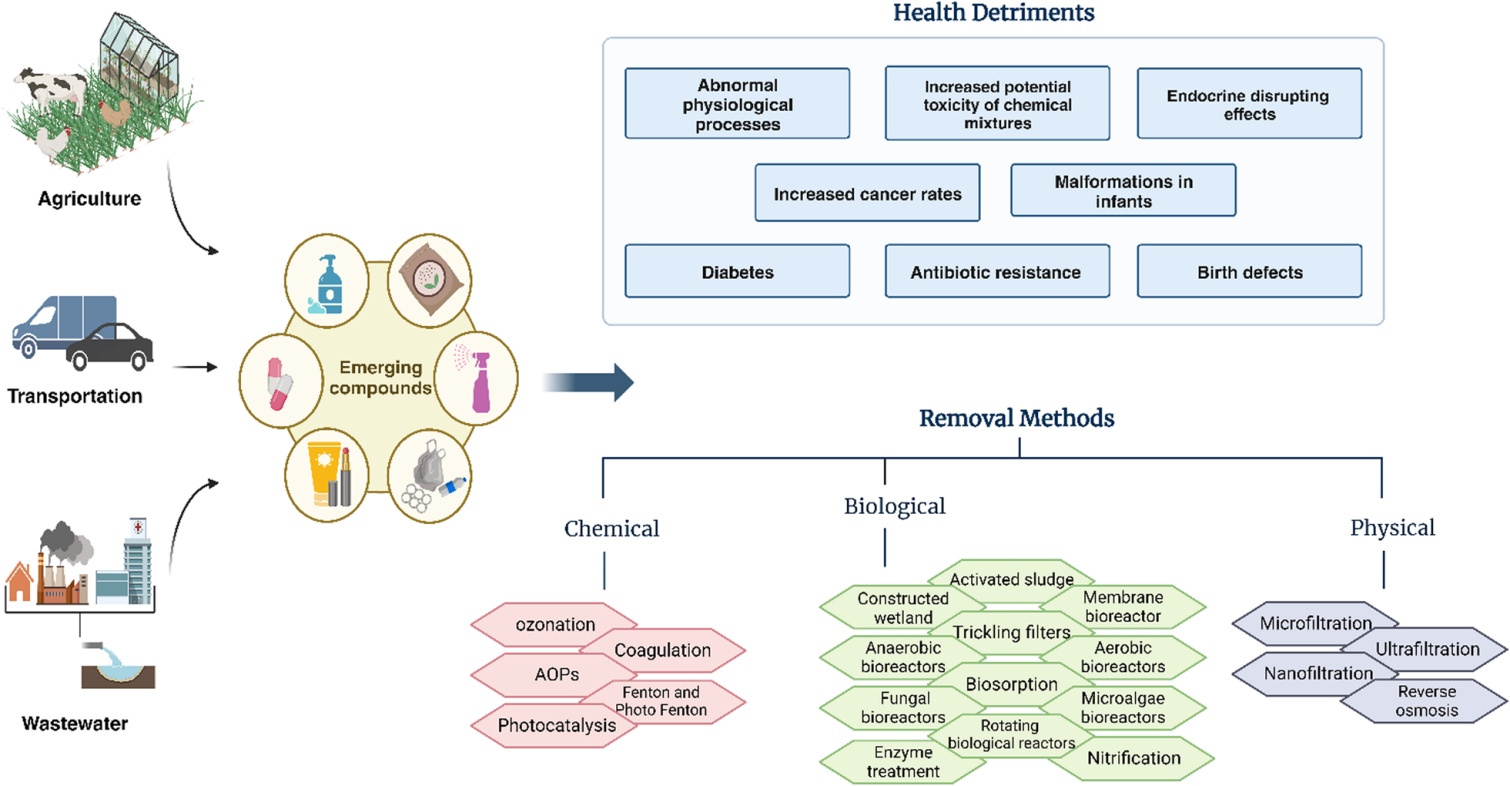
Sources, hazards, and available removal methods of emerging compounds, including physical, chemical, and biological approaches. AOPs: Advanced oxidation processes. Created with BioRender.com

Among various conventional, non-conventional, chemical, and physical treatment processes [9, 10], bio-based remedial techniques (see Fig. 1 for more information) are known for being safer, more cost-effective, and require less energy than physicochemical techniques [11, 12]. Bioremediation and biodegradation methods have the added benefit of being eco-friendly as they involve the interaction and cooperation of microbial consortia to remove contaminants [13, 14]. However, these interactions are complex and unpredictable, since more than 99% of microbial cells are difficult to culture using traditional techniques [13]. To address this challenge, the systems biology approach that combines geochemical data with biologically pertinent measurements can be used. This approach can detect contamination sources and propose remedial methods. It relies on biological materials that act as "biosignatures," such as genomes, proteins, lipids, metabolites, and transcribed RNA [15, 16]. Omics-based studies that track these biosignatures are promising approaches giving a broad vision of biodiversity in microbial communities and their potential to remove persistent pollutants. Based on their enzymatic profile and/or their expressing genes, these methods employ innovative tools for discovering the hidden worlds of microorganisms and their potential. This target would be achievable by taking advantage of next-generation sequencing techniques along with bioinformatics, and genomic/RNA/ microbial metabolic pathways/proteins databases that are the main tools of omics approaches [17, 18]. The use of omics methods has grown in importance for understanding and improving microbial-facilitated remediation of environmental pollutants. Genomics, transcriptomics, proteomics, and metabolomics offer valuable insights into the microbial populations engaged in remediation and the underlying molecular processes [19]. These advanced techniques have empowered researchers to more comprehensively characterize the composition, operation, and changes of microbial communities in contaminated settings. Applying omics methodologies has facilitated the identification of new microbes and enzymes for bioremediation, as well as the enhancement of existing microbial communities for more effective contaminant elimination [20]. This, in turn, aids in pinpointing the crucial microbes and enzymes responsible for breaking down pollutants. Studies based on omics have revealed previously unknown microbial pathways and genes crucial to the degradation of diverse environmental toxins such as pesticides and industrial chemicals [19, 21, 22].

Soil contains a wide variety of microbial communities with many potential remedial properties, some of which have been discovered while others remain unknown. The absorption of pollutants by plants from the soil can lead to harm in ecosystems as they move up the food chain [23–25]. The presence of different microorganisms in the soil drives the process of bioremediation, leading to the breakdown of contaminants through the interactions of a community of microbes. The effectiveness of bioremediation relies on the capacity of these microorganisms to adjust to changing environmental circumstances [26]. In this regard, culture-independent omics-based approaches are the key methods to unlock the undetected side of this potential. Despite the fact that there are reviews related to soil bioremediation, there is still a need to investigate the potential of soil microbial populations in breaking down emerging organic contaminants with the help of novel and more accurate omics approaches. So, in this review, we aim to address the lack of information on using omics approaches to remediate soils contaminated with emerging organic pollutants and the way through the future of this field.

## Emerging organic contaminants: a threat to the environment and biota

Emerging organic contaminants (EOCs) and their metabolites, which are frequently more toxic than their initial source, have been found in a variety of environments [27]. Emerging organic contaminants (EOCs) do not have established environmental monitoring or emission standards and can have harmful effects on ecosystems and human well-being. Because of their potential risks to the environment and human health, continuous release into the environment, and the challenge of fully removing these substances even with advanced wastewater treatment plants (WWTPs), EOCs may require future regulatory attention. Recent advancements in environmental analysis have resulted in frequent detection of these substances in various settings such as sewage, surface water, drinking water, and soil [28].

In vivo studies have shown that exposure to EOCs can cause hormonal imbalance, decreased aquatic organism survivability, reproductive issues, and a variety of health problems in humans, including cancer, diabetes mellitus, respiratory disorders, neurological disorders, metabolic diseases, and thyroid disease [29–31]. This is a cause of concern as some EOCs like Perfluorooctanoic Acid (PFOA) and Perfluorooctanesulfonate (PFOS) have been found in wildlife, drinking water, human serum, and breast milk [29]. Additionally, EOCs can be harmful to other living beings such as birds, fish, insects, and non-target plants [30]. Generally, based on scientific research, the immediate and long-term impacts of these environmental contaminants on ecosystems, natural resources, human health, and the environment have been proven [32] (Table 1).

**Table 1:** A summary of emerging organic compounds list, their sources, and toxic effects [29, 30, 33–39]

Number	Name of compounds	Source	Toxic effects on living beings
1	Organochlorine (diazion, endosulfan, pentachlorophenol)	Pesticides	Diarrhea, blurred vision, respiratory disorder, blood problems such as aplastic anemia in humans, cardiovascular disease in rats and mice
2	Carbamates (carbofuran, thiourea)	Insecticides, nematicides, acaricides, rodenticides	Hypertension, vision defect, respirator disorder, bone marrow damage, carcinogenic effects in humans
3	Pyrethroids (tetramethrin, decamethrin)	Insecticide	Mutagenic effect, neurotoxin, paraesthesia in humans
4	Carbanilates (chlorpropham, diuron)	Herbicide	Kidney and liver failure, anemia, and bone marrow damage in humans
5	Acetamides (diphenamid)	Herbicide	Dermal irritation, inhalation toxicity in humans
6	Phenoxy allocates (2,4-dichlorophenoxyacetic acid)	Herbicide	Nervousness, headache, dizziness in humans
7	Triazines (cyprazine, propazine)	Herbicide	Constipation, nausea, dizziness, anemia in humans
8	Benzoic acid derivatives (dicamba)	Herbicide	Damage to CNS, heart failure in humans
9	Benzonitriles (bromoxynil)	Herbicide	Vomiting, urinary disorder in humans
10	Phthalimides (folpet)	Fungicide	Brain ailment, skin disease in humans
11	Detergents (alkylphenols)	Industrial and domestic	Respiratory, vision, and movement disorders in fish, disturbance in estrogen production and reproduction with increased number of eggs produced by minnows and vitellogenin level
12	UV filters (benzophenone-3 (BP-3), 3-(4-methylbenzylidene) camphor (4MBC), ethylhexyl methoxycinnamate (EHMC), and octadecene (OC)	Personal care products	Can bind to human hormone receptors and have binding or antagonistic effects on hormones, endocrine-disrupting influences (disruption of the hypothalamic-pituitarythyroid axis (HPT) and reproductive and developmental function) in lab animals
13	Endocrine-disrupting chemicals (EDCs) (steroid estrogens (SEs), PCBs, OCPs, dioxins)	Industrial chemicals, pharmaceuticals, personal care products, herbicides, and pesticides	Feminization in fish, reproductive abnormalities in birds, obesity, diabetes, various cancer types, cardiovascular risks, metabolic disorders, epigenetic alterations, autism in humans
14	Phthalates	Plasticizers	Obesity-related factors, glucose disturbances, hypertension in humans, reduced levels of thyroid hormones and progesterone in pregnant mothers
15	Bisphenol A (BPA)	Plasticizers	Estrogenic effects in rats, possible EDC in birds, hormonal effects in humans and animals, xenoestrogen, possible carcinogenic effect in humans
16	Quaternary ammonium compounds (QACs)	Disinfectants, fabric softeners, preservatives, and cosmetics	Reduced fertility in mice, respiratory issues such as asthma, increased risk of chronic obstructive pulmonary disease (COPD), neurological defects, decreased mitochondrial function in humans
17	Nonsteroidal anti-inflammatory drug (diclofenac)	Pharmaceuticals	Reduces the hematocrit values of fishes, and causes cytological changes in the liver, kidneys, and gills of fishes
18	β-blocker (propranolol)	Pharmaceuticals	Reduction of viable eggs of Japanese medaka (Oryiaslatipes), strong acute toxicity on benthos and zooplankton
19	Antibiotics (penicillin, sulfonamides, tetracyclines)	Pharmaceuticals	The development of resistance in bacterial pathogens
20	Acetaminophen	Pharmaceuticals	Disruption of the steroidogenic pathway in humans, chronic hepatotoxicity (cirrhosis and hepatocyte necrosis) in mice

## Bioremediation: a convenient way to remove EOCs from the soil

For the contaminated soils, sediments, and water remediation, bio-based removal techniques such as phytoremediation and microbial-based bioremediation/biodegradation offer a financially advantageous and eco-friendly alternative to conventional physicochemical treatments that combined with biostimulation and bioaugmentation techniques speed up microbial activities in polluted sites [40, 41]. The type of pollutant, environmental conditions, and accessibility of phosphorus and nitrogen supplies all impact biodegradation efficiency [42, 43]. Moreover, factors including the type of microorganisms used, the screening situation, and the genetic profile of the organisms can affect microbial activity [44]. Even though biodegradation is considered a safe and eco-friendly technology to remove synthetic chemicals from the environment, it is important to consider that the process should not introduce more harmful substances into the environment than there originally were [45].

Microbial bioremediation refers to using microorganisms or their byproducts, such as enzymes (including cytochrome P450, laccases, hydrolases, dehalogenases, oxygenase, dehydrogenases, proteases, transferases, oxidoreductases, and lipases) [46–48], or their leftover biomass, to remove contaminants from the environment. Oxidoreductases and hydrolases are the two groups of enzymes with high biodegradation activity [49]. The former is responsible for detoxifying toxic organic compounds through oxidative coupling. This leads to the breakdown of chemical bonds and the transfer of electrons via oxidation–reduction reactions, resulting in the oxidation of contaminants to harmless substances. Oxidoreductases and peroxidases are present in bacteria, fungi, and higher plants [49, 50] and could play a role in the decomposition of lignin and the humification of phenolic and aromatic substances in soil [51, 52]. Additionally, they can detoxify toxic xenobiotics by polymerization, copolymerization, or binding to humic substances [50, 53]. Laccases belonging to oxidoreductase enzymes are powerful oxidizers of pesticides, PhACs, and hormones that could be purified from white-rot fungi [54, 55]. *Pleurotus ostreatu*s as an example could remove bisphenol A. Their potential to degrade persistant compounds is mainly related to lignolytic enzyme production, making them xenobiotic-tolerant microorganisms. Monooxygenase as an oxidoreductase enzyme integrates oxygen atoms with the substrate in the reduction reactions and also performs hydroxylation, denitrification, ammonification, dehalogenation, and sulfurization of the substrate [50]. *Buttiauxella* sp. S19-1 as an example is a TNT-degrading bacterium with oxydoreductase activity [56]. In addition, hydrolytic enzymes play a role in disrupting chemical bonds of toxic compounds, and reducing their toxicity [49, 50]. This makes them effective agents for biodegrading oil spills, organophosphates, and carbamate pesticides. Hydrolases catalyze condensation and alcoholysis reactions. They are advantageous due to their availability, lack of cofactor stereoselectivity, and tolerance to water-miscible solvents. Lipase is an enzyme from the group of hydrolases that plays a role in the decomposition of organic oil pollutants, and its mechanism is the conversion of triacylglycerols into glycerol and free fatty acids [49]. Eventually, when contaminants reach the metabolic pathways for degradation and biotransformation, microorganisms can degrade them via their own or modified metabolic processes [49].

Despite several reports that focused on single strains' ability to biodegrade pollutants, microbial communities or consortiums of microbial strains are known to be more effective in remediation, especially in natural conditions. This is due to their co-metabolism that lessens cross-reactions and the metabolic pressure on single strains of the community [57]. Because of the cooperation of diverse species in consuming a compound as a substrate, microbial consortia frequently operate better and are more resistant in polluted settings; as a result, they are more efficient at bioremediation than particular single-strain microorganisms [17, 58]. Both bacterial and fungal intracellular and extracellular enzymes are being used to remediate the resistant contaminant [59, 60]. Due to the fact that bacteria grow more quickly and fungi produce stronger enzymes, fungal and bacterial consortia typically outperform single-strain cultures in their ability to break down resistant contaminants [61, 62]. As an example of the decolorization of a single dye, the bacterial and yeast consortium (*Brevibacillus laterosporus* and *Geotrichum candidum*) produced faster decolorization rates than the individual microbiological species [63]. Studies have shown that the joint activities of enzymes of mixed microbial cultures are much better in removing some pollutants compared to individual strains [63] which is why microbial populations in the consortia structures are promising organizations for biotreating degradation-resistant pollution.

However, it is important to ponder the drawbacks and risks associated with bioremediation for a more effective utilization of this method. These concerns include the comparably slow pace of the process in comparison to other remediation technologies, as well as the limitation in completely removing all amounts of contaminants. It is important to note that this method may not be suitable for cleansing certain mineral pollutants or organic compounds. Additionally, there are challenges in confirming the complete elimination of contaminants. Furthermore, during the decomposition of toxic compounds, there is a possibility of generating more toxic byproducts. On the other side, during bioremediation processes, there is a possibility that organic nutrients like animal manure and sewage sludge might carry antibiotic residues and resistant bacteria. Antibiotic resistance has the ability to disseminate globally through horizontal gene transfer, influencing both targeted and non-targeted microbial communities. The transfer has the potential to contribute to the rise of antibiotic-resistant strains and may impact human health by diminishing the efficacy of antibiotics in treating bacterial infections. These limitations are therefore crucial considerations when assessing the use of bioremediation for specific contaminated sites [64–66].

Some microorganisms and their effective enzymes in the biodegradation of emerging organic compounds are listed in Table 2.

**Table 2:** EOC-degrading microorganisms and enzymes

Microorganism	Enzyme	Compound	References
*Schizophyllum commune* IBL-06	Lignin peroxidase	Diclofenac	[49, 67]
*Trametes versicolor* and *Pycnoporus sanguineus* CS43*, Pleurotus ostreatus*	Laccase	Bisphenol A, PCBs (polychlorinated biphenyls)	[49, 68–70]
*Nocardioides sp.* C190*, Pseudomonas, Rhodococcus erythropolis*	Atrazine dechlorinase, triazine hydrolase	Triazine herbicides	[68, 71, 72]
*Aspergillus niger* NCIM 563	Phytase	Organo phosphate	[68, 71]
*Pseudomonas sp.* LBr	Glyphosate oxidase (GOX)	Glyphosate (pesticides)	[43, 73]
*Pleurotus* sp*.*	Laccase, manganese peroxidase, lignin peroxidase	Degradation of Azo dyes	[74]
*Ganoderma lucidum*	Laccase, manganese peroxidase, lignin peroxidase	Degradation of phenanthrene and pyrene	[74]
*Stropharia coronilla*	Manganese peroxidase	biodegradation of benzo(a)pyrene	[74]
*Phanerochaete chrysosporium*	Peroxidases (Lignin peroxidase & manganese peroxidase)	Degradation of pesticides (atrazine & alachlor), nitroaromatic compounds	[71, 74]
*Trametes versicolor*	Laccase	Degradation of herbicide isoproturon, anthracene, and benzo(a)pyrene	[71, 74]
*Bacillus subtilis*	Laccase & Esterase	Biodegradation and detoxification of Cypermethrin (insecticide), Bisphenol A (BPA)	[74, 75]
*Trametes versicolor*	Laccase	Degradation of Carbofuran	[74]
*Candida antarctica* (CAL), *Candida rugosa* (CRL)	Lipases	Poly (bisphenol-A carbonate) (BPAPC)	[76]
*Bacillus subtilis, Bacillus pumilus, Chromobacterium viscosum,* and *Sphingobacterium sp.* strain S2	Lipase	PBSA, PLA, PCL, oil, and PBS	[77]
*Pseudomonas aeruginosa* PA1	Carboxylesterases	Malathion and parathion (insecticide)	[71]
*Humicola* sp.	Cellulase	Detergent and washing industrial contaminants	[71]

## The way through discovering the composition and function of EOC-degrading microbial populations: omics approaches

**Fig. 2:**
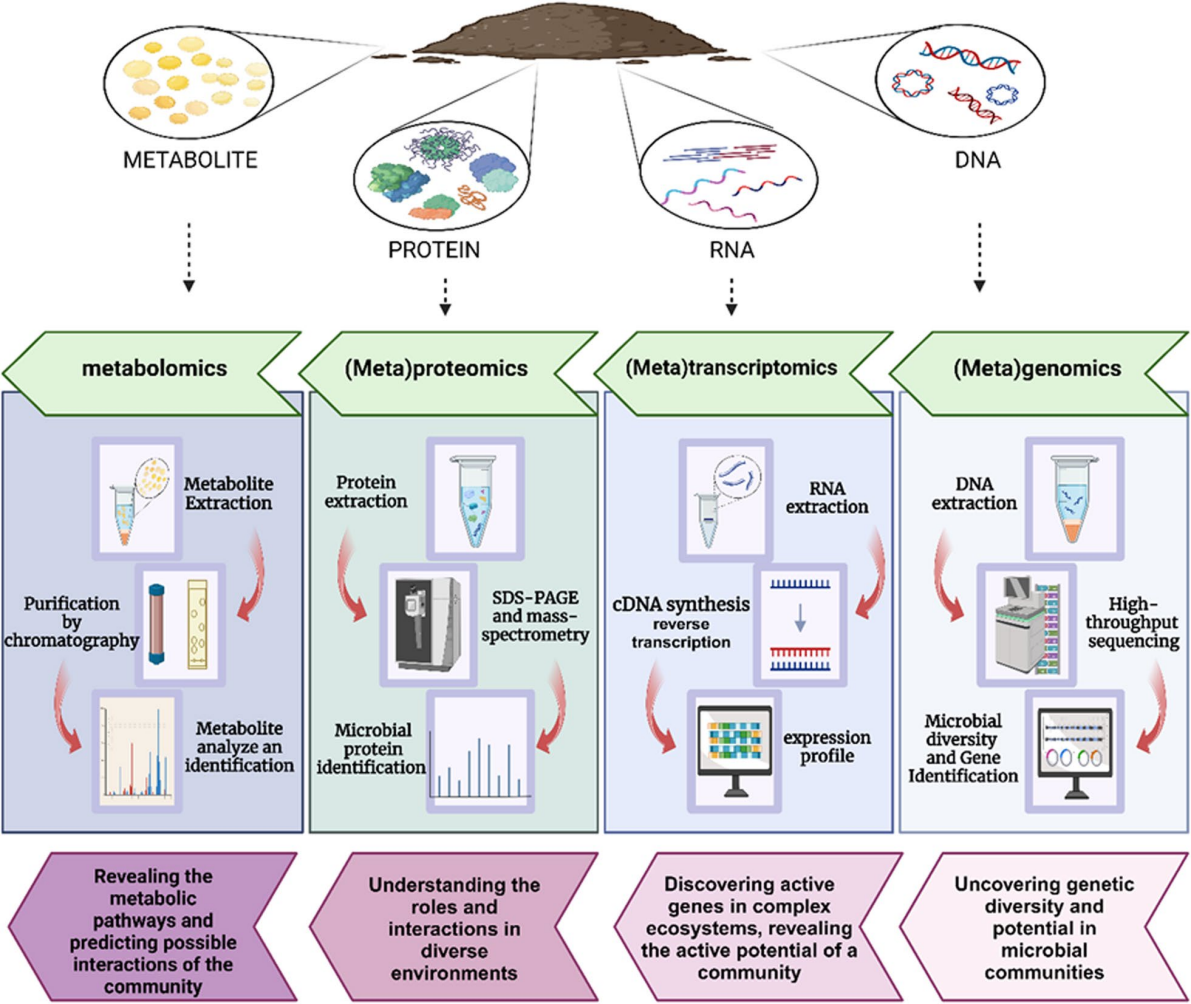
Available omics-based methods to discover emerging pollutants' remedial potential in the soil microbial communities. Each approach has its standard procedure and available techniques, giving different information from diversity to the metabolic potential of the microbial communities. Created with BioRender.com

To understand the soil ecosystem, it is necessary to discover its microbial population, activities, and how they interact with the soil compartments [78]. This vision in a specific ecological niche has been given more weight by molecular approaches such as genomics, proteomics, transcriptomics, metabolomics, fluxomics, etc. [79]. The gathered data from different "omics" methodologies is then refined to offer adequate in-depth information to study microbial metabolism in bioremediation and provide full knowledge of the soil microbial population, their functional and key genes, mechanism of toxicity, and interaction details. [78, 80] (Fig. 2). Applying omics techniques, it is possible to investigate the changes in expression profiles associated with the degradation of compounds (proteomics), to identify and quantify specific metabolites that arise during degradation (metabolomics), and changes in the gene expression that accompany the exposure of microorganisms to pollutants (transcriptomics) [16].

Omics approach can reveal the specific genetic determinants, metabolic pathways, and regulatory mechanisms that enable certain microbes to thrive in contaminated conditions. This knowledge can guide the selection and engineering of more effective inoculant strains [81, 82]. Omics can also elucidate how introduced inocula interact with the native microbiome, including competition, cooperation, and succession processes that impact colonization success. This informs strategies to better integrate inocula into the existing community [82, 83]. Omics can reveal the specific abiotic and biotic stressors in contaminated sites that limit inoculant survival and proliferation, guiding the development of more stress-tolerant strains [83].

Genomics and metagenomics provide insights into the genetic makeup and metabolic capabilities of microbial communities involved in bioremediation [84, 85]. This allows researchers to identify microbes with desirable traits for degrading pollutants, tolerating harsh conditions, and thriving in contaminated environments [86, 87].

Transcriptomics and proteomics reveal the genes and proteins that are actively expressed by microbes during bioremediation, shedding light on the molecular mechanisms underlying pollutant degradation and stress responses. This knowledge can guide the engineering of more effective bioremediation strains or consortia [85, 88]. Metatranscriptomics and metaproteomics can track the activity and expression of target inoculant strains within the complex environment, allowing researchers to evaluate and optimize their performance [81, 89].

Metabolomics profiles the small molecule byproducts of microbial metabolism, tracking the flow of carbon, energy and nutrients during bioremediation. This helps optimize nutrient amendments, electron donors, and other environmental conditions to maximize microbial activity and pollutant removal [84].

Integrating multi-omics data provides a systems-level understanding of plant–microbe interactions, microbial community dynamics, and the complex biological processes involved in bioremediation. This enables predictive modeling and rational design of robust, efficient microbial inoculants and bioremediation strategies [85].

### Role of metagenomics in the identification of effective EOC-degrading microorganisms/genes

The systematic study of microbial communities at the genome level is called metagenomics. Over 99% of microorganisms that live in various natural habitats are either difficult or impossible to cultivate, leading to major limitations on processes that rely on culture. This method makes it possible to explore a sample's entire genome sequence (genomics) and an enormous amount of genome that is straightly extracted from the environment (metagenomics) [90].

The following steps could be taken to acquire metagenomics data [91–93], including:

I. Sampling and processing: Samples should be representative and appropriate DNA extraction methods should be used.
II. Sequencing technology: Metagenomic sequencing has shifted from Sanger sequencing to next-generation sequencing (NGS) technologies, such as the 454/Roche, Illumina/Solexa systems, Pyrosequencing, PacBio, and Ion torrent sequencing.
III. Assembly: Short-read fragment assembly is used to construct longer genomic contigs through co-assembly and de novo assembly methods.
IV. Binning: Organizing assembled contigs into collections based on their probable taxonomic or functional classifications. This step is substantial for data analysis and finding key players and/or genes in the community.
V. Annotation: Assigning functions to genes or gene products based on their similarity to known proteins or gene products in databases.
VI. Statistical analysis: Measurement of alpha and beta diversity and the detection of differentially abundant taxa or functions, could be interpreted from metagenomic data outcomes using statistical analysis.
VII. Data storage and sharing: To ensure that the findings of various investigations can be compared and contrasted and in order to develop metagenomic datasets, the extracted data should be deposited into databases.

The metagenomic approach with pure DNA can take two paths: (1) bioactive compounds screening in clones of the metagenomic library which is now obsoleted (function-based) [94–97] and (2) complete genomic DNA sequencing (sequence-based). The extracted data of metagenomics contain information on the microbial communities, including their diversity and taxonomic characterization at the community level and their functional and metabolic potential. Predicting the community’s functional capabilities and nutritional requirements provides the key insights needed to formulate selective media that will support the growth of these elusive microorganisms [94, 98–100].

Metagenomics has identified biomolecules such as antibiotics and microbial enzymes. It also helps to explore the hidden potential of bioremediation-relevant microbes [78]. Genomic-based identification of new promoters, genes, and degradative pathways has helped to develop more efficient pollutant-degrading strains for bioremediation [101]. Key bioremediant bacterial genera like *Pseudomonas*, *Shewanella*, *Deinococcus*, and *Dehalococcoides* have their whole genome sequences available, and detecting novel genes in such strains could provide insights into their degradation ability and substrate selectivity [102].

By analyzing the whole genome of a biomass sample, it is possible to identify the different levels of microbial taxonomy and pathways for breaking down xenobiotic compounds, both aerobically and anaerobically [103]. Garrido-Sanz, D. et al. (2018) isolated and analyzed a Polychlorinated Biphenyls (PCBs) biodegrading bacterial consortium through 16S rRNA amplicon and whole genome shotgun sequencing. *Pseudomonas* and *Rhodococcus* strains were abundant in this consortium, harboring enzymes that catalyze biphenyl to benzoate and benzoate to Tricarboxylic Acid (TCA) cycle intermediates. The study demonstrated that metagenomic analysis can identify bacteria and their specific reactions and pathways involved in biodegradation processes [104]. The possible potential of *Bacillus, Krasilinkoia, Lysinibacillus, Rhodococcus, Sphingobium, Rubrivivax, Paenibacillus,* Nitrate Reducers, and *Enterobacter* species in remediation of azithromycin-contaminated soil was also another achievement of this approach [105]. *Pseudomonas*, *Achromobacter*, *Xanthomonas*, *Stenotrophomonas*, and *Cupriavidus* were found to be major players in atrazine bioremediation in the study of Bhardwaj et al. (2019) using whole metagenome sequencing [106]. *Streptomyces nigra* LM01 is a cornfield-isolated strain that also efficiently degrades atrazine and nicosulfuron. *atzA*/*trzN* were identified in its whole genome, indicating its possible potential to dechlorinate atrazine to hydroxyatrazine or convert it to cyanuric acid [107]. In another study based on a metagenomic fosmid library, the ability of soil microorganisms in polycyclic aromatic hydrocarbon degradation was assessed. Extradiol dioxygenases encoding genes and Rieske non-heme iron oxygenases were identified in a complex microbial network. These enzymes are responsible for activating aromatic compounds by substituting methyl groups to their side chains [108].

Microbial degradation is regarded as the most appropriate technique for degrading chlorimuron-ethyl, a common long-term residual sulfonylurea herbicide. *Rhodococcus erythropolis* D310-1 is a strain that harbors CarE, participating in the catalysis of chlorimuronethyl de-esterification through the catalytic action of carboxylesterase. This provides new insights into the process of sulfonylurea herbicide degradation as well as the theoretical datasets for enzymes [109]. Organophosphates (OPs) such as chlorpyrifos (CP), are another type of pesticide that impairs soil fertility and disturbs the biogeochemical cycle. *Pseudomonas aeruginosa* RNC3 and *Stenotrophomonas maltophilia* RNC7 are common soil bacteria that have significant CP breakdown potential. The genomes contain annotations for degradation processes and metabolism of aminobenzoate, chlorocyclohexane, chlorobenzene, toluene, and naphthalene. Organophosphorus hydrolase and 4-nitrophenol 4-monooxygenase are the major enzymes of the aminobenzoate degradation pathway which play important roles in CP breakdown. Furthermore, results demonstrated that phenolic compound oxidation is the most important step of CP biodegradation. Together with *opd* and *opch2* genes, a series of putative CP degradation genes were expressed in RNC3 and RNC7 including metallophosphoesterase (OPCH2) and phosphotriesterase encoding genes [110].

In a recent study, Cao and colleagues (2024) discovered a novel *Klebsiella pasteurii* strain capable of degrading the herbicide mesotrione. This strain exhibited exceptional adaptability to environmental conditions. Through genomic analysis and RT-qPCR, the researchers identified the nitroreductase-encoding genes *nfsA* and *nfsB* as key players in mesotrione biodegradation, a finding that had not been reported before [111]. In another study, Sun et al. (2024) used diethyl terephthalate (DET) as a screening substrate to discover a new amidohydrolase gene, AmiH52, in a soil metagenomic library. The recombinant enzyme, expressed in *E. coli*, demonstrated both esterase and amidohydrolase activities. It exhibited highly specific activity towards p-nitrophenyl butyrate and the ability to degrade various amide herbicides. AmiH52 was found to be particularly effective against the herbicide propanil, showing the most potent degradation activity [112].

Table 3 introduces the potentially EOC-degrading microbial cells and consortioms, discovered through genomic or metagenomic-based approaches in past years.

**Table 3:** EOC-degrading microbial communities that have been identified through genomic/metagenomic approaches

Microorganism/Microbial consortia	Method and Platform	Contaminant	References
*Sulfuricurvum* spp*., Pseudomonas* spp*.,* and* Candidatus saccharibacteria*	16S rRNA/Illumina MiSeq	Hydrocarbons and their derivatives	[113]
*Rhodococcus, Sphingomonas,* and* Pseudomonas*	Shotgun metagenome sequencing/Illumina HiSeq	HCH, Endosulfan, and DDT pesticides	[114]
*Acinetobacter johnsonii* LXL_C1	Whole genome sequencing/Illumina HiSeq and PacBio	Cyprodinil	[115]
*Clostridium, Nocardioides, Bellilinea, Anaerolinea, Longilinea,* and* Phycicoccus*	16S rRNA/Illumina Miseq	Cypermethrin, fipronil, imidacloprid, and sulfosulfuron	[116]
*Geobacter, Mycobacterium,* and* Sphingomonas*	16S rRNA/Ilumina MiSeq	Polycyclic aromatic hydrocarbons (PAHs)	[117]
*Cellvibrio bacteria, Acidobacteria* (such as *Candidatus Koribacter* and *Candidatus Solibacter*)	16S rRNA/Illumina Miseq	Acetaminophen (APAP)	[118]
*Pseudomonadaceae, Rhizobiacaea, Desulfobacteriacea, Deinococcaceae, Bacillaeace, Sphingiomonadaceae, Xanthomonadaceae,* and* Enterobacteriaceae, Paneibacilleacea, Bradyrhizobacea*	16S rRNA/IonTorrent	Azithromycin	[105]
*Pseudomonas mendocina, Brevundimonas olei, Serratia marcescens, Sphingomonas*	16S rRNA/Illumina Miseq	Polycyclic aromatic hydrocarbons (PAH)	[119]
*Acinetobacter* spp*.*	Metagenomic libraries (BAC and fosmid clone DNAs)/Illumina HiSeq	Toluene	[120]
*Sphingobium fuliginis* ATCC 27551	Whole genome sequencing/Pacbio RSII and Illumina Miseq	Neurotoxicorganophosphate insecticides	[121]
*Nocardioides carbamazepini* sp*.* nov	Shotgun metagenome sequencing/Illumina MiSeq	Carbamazepine and ibuprofen	[122]
*Thermobifida fusca, Pseudomonas mendocina,* and *Nocardia* sp*.*	Shotgun metagenome sequencing	Different types of plastic wastes	[123]
*Actinomycetales, Gemmatimonadetes, Proteobacteria, Acidobacteria,* and* Bacteroidetes*	Shotgun metagenome sequencing/Illumina HiSeq	Di(2-ethylhexyl) Phthalate (DEHP) (a plasticizer)	[124]
*Pseudomonas, betaproteobacteria and Rhodococcus, Bordetella, Stenotrophomonas* sp*., Achromobacter and Variovorax*	16S rRNA/Shotgun metagenome sequencing/Illumina MiSeq	Polychlorinated Biphenyls (PCBs)	[104]
*Proteobacteria, Acidobacteria, Actinobacteria, Chloroflexi, Firmicutes,* and* Gemmatimonadetes*	16S rRNA/Illumina Miseq and Illumina Hiseq	Perfluorinated Compounds (PFCs)	[125]
*Bradyrhizobium, Mycobacterium, Rhodopseudomonas, Pseudomonas, Cupriavidus,* and *Streptomyces, Rhodococcus, Starkeya, Rhizobium, Sphingomonas, Ochrobactrum, Methylobacillus, Alicycliphilus* and* Stenotrophomonas*	Shotgun metagenome sequencing/Illumina HiSeq	Carbamazepine (CBZ), triclocarban (TCC), and triclosan (TCS)	[126]

### Transcriptomics and metatranscriptomics: identification of the active EOC-degrading genes

In order to understand the functional activities of the soil microbial communities, it is necessary to study transcription gene profiles, named "transcriptomics or metatranscriptomics" [17, 127]. The analysis of mRNAs allows for a clear understanding of gene expression in specific cells and tissues (Fig. 2). This includes determining the presence or absence of transcripts, evaluating alternative splicing to predict protein isoforms, and quantitatively assessing how genotype influences gene expression through the analysis of expression assessable trait loci or allele-specific expression [79]. While transcriptomics examines the gene expression profile of a single organism at a particular growth stage, metatranscriptomics examines this profile for the microbial community by extracting RNA from an environmental sample. The best procedures would involve complete mRNA extraction and enrichment, cDNA synthesis, microarray hybridization of cDNA, RNA-Seq, and reference mapping of sequence reads. The most important step is selective mRNA enrichment by rRNA depletion, followed by mRNA transcript sequencing [128, 129]. The two main techniques used nowadays to determine the transcriptional profile for virtually every biological sample under a wide range of circumstances are microarrays and RNA-Seq [130]. Using this approach and based on the Illumina HighSeq2500 sequencing method, Sharma et al. (2019) indicated that Archaea play a more substantial role in the nitrification process in metal and pesticide-contaminated soil than bacteria, demonstrating their active role in contaminated environments. This domain displays strong expression of transcripts for the glyoxalase and/or bleomycin resistance dioxygenases, 4-hydroxyphenylpyruvate dioxygenase, 2-nitropropane dioxygenase, metapyrocatechase, ring hydroxylating dioxygenases, and intradiol dioxygenase (from *Novosphingobium* spp.) genes related to aromatic hydrocarbon degradation in agricultural soil [131]. Understanding the key metabolic pathways involved in the biodegradation of xenobiotics and EOCs is a significant area of research in molecular biology. Studying the metabolic pathways in the biodegradation of chlorimuron-ethyl by *Rhoococcus erythropolis* D310-1 revealed toluene and aminobenzoate degradation as key pathways. During this process, essential genes like carboxylesterase, cytochrome P-450, and glycosyltransferase were identified through qRT-PCR experiments [132]. Brzeszcz et al. (2020) investigated the potential of seven non-pathogenic bacterial strains from the genera *Rhodococcus*, *Mycolicibacterium*, *Dietzia*, *Pseudomonas*, *Arthrobacter*, and *Gordonia* as bioaugmentation agents in soil historically contaminated with aliphatic and polycyclic aromatic hydrocarbons. They evaluated the effects of biostimulation and bioaugmentation on the transcriptomic profiles of the soil. The study found that *Gammaproteobacteria* and *Actinobacteria* classes were associated with alkane monooxygenase (AlkB) transcripts, with a significant proportion attributed to *Pseudomonas* and similarities to genes from *Mycobacteriaceae*, *Gordonia*, and *Arthrobacter* genera [133]. Comparing and analyzing the microbial community of biobed systems before and after a pesticide usage season in the field, Russell et al. (2021) showed a significant rise in the aromatic and xenobiotic degradation-related genes, including peroxidases, monooxygenases, and cytochrome P450. Metagenomic and metatranscriptomic analyses encourage approaches in which pesticides are removed in biobeds as the result of a complex network of interacting biodegrader activities that are highly enriched with bacteria: *Pseudomonas, Sphingobium,* and *Oligotropha*. Sharma et al. (2017) also conducted a metatranscriptome analysis on agricultural soil that has been exposed to chemical fertilizers and pesticides for many years, estimated to be polluted with heavy metals. It was discovered that the ecosystem contains a variety of organisms, with bacteria being the most prevalent, including *Achromobacter, Pseudomonas, Bacillus, Sphingobium, Micrococcus, Serratia, and Streptomyces* species. A high abundance of aromatic dioxygenase transcripts, related to breaking down Catechol, Benzoate ring, and Gentisate was also found in the sample [134]. Through metatranscriptomic research, it has been revealed that pesticide treatment and exposure to other EOCs will alter the microbial diversity of the soil. How this alteration will affect the functionality of the microbiota is the role of metatranscriptomic analysis [135]. Reducing the abundance of *Mesorhizobium*, *Rhodopseudomonas*; and *Stenotrophomonas*, a genus known to break down xenobiotics is one of the findings of this approach in this subject [136].

Metatranscriptomic could also be a tool to comprehend the microbial communities and their capacity to degrade organic contaminants in the soil. In this way, Singh et al. (2018) looked at the metatranscriptome data of wheat rhizosphere samples. According to their analysis, a total of 118 transcripts belonging to 47 distinct enzymes associated with 21 pathways involved in the breakdown of aromatic compounds. The abundance of aromatic amines degradation-related transcripts, and those related to carbazoles, benzoates, and naphthalene degradation, the ketoadipate pathway, phenols, biphenyls, and xenobiotics removal in the soil samples suggest that these substances can be degraded effectively. 2-hydroxy-6-oxo-6-phenylhexa-2,4-dienoate hydrolase is a specific enzyme, frequently found in various aromatic-degrading metabolic pathways, including carbazole, biphenyl, and central meta-cleavage pathway. The taxonomic analysis showed that the predominant communities that play a role in the degradation of aromatic compounds were bacteria, particularly *Proteobacteria, Actinobacteria, Firmicutes, Bacterioidetes,* and *Cyanobacteria*. These findings imply that the soil's aromatic contaminants are significantly removed by microbial communities connected to crop rhizospheres [137]. While being the key players, microorganisms are not the only sectors of the bioremediation and biodegradation process, especially in the soil with intricate interactions between microorganisms and plants. Metatranscriptomics in this regard, could be used to decode these interactions that are the driving force of resistant pollution removal. In other words, co-metabolism will increase the efficiency of bioremediation in many cases. Focusing on the collaboration between plants and microorganisms using tripartite metatranscriptomics, Tartaglia et al. (2022) conducted a mesocosm metatranscriptomic study on the rhizospheric soil containing *Festuca arundinacea* roots. The occurrence of extraction, degradation, and metabolism of xenobiotics detected in contaminated soil illustrates the phytoremediation process carried out through a tripartite activity involving plants, bacteria, and fungi. The presence of *Actinobacteria* and fungi has been found to be high, which could have an effect on the success of the remediation process. Some transcripts have been linked to PAH degradation including laccase, monooxygenase, and peroxidase. These are protocatechuate 3,4-dioxygenase, protocatechuate 4,5 dioxygenase, salicylate 1-monooxygenase, naphthalene 1,2 dioxygenase, and 4,5-dihydroxyphthalate decarboxylase. The majority of transcripts associated with protocatechuate 3,4-dioxygenase and protocatechuate 4,5-dioxygenase were from *Actinobacteria*, particularly the *Nocardioides* and *Streptomyces* genera [138]. deMenezes et al. (2012) discovered a grown concentration of transcripts linked to dioxygenase, stress response, and detoxification as a result of phenanthrene exposure in soil microbial communities [139]. Willow rhizospheres cultivated in contaminated soils were notably supplemented in transcripts related to PAH degradation, mainly belonging to the orders *Actinomycetales, Rhodospirillales, Burkholderiales, Alteromonadales, Solirubrobacterales, Caulobacterales, and Rhizobiales* [140, 141]. The response of *Arthrobacter* QD15-4, isolated from plastic-contaminated soils to dimethyl phthalate (DMP), a prevalent environmental pollutant, was studied by Wang et al. (2019). This strain demonstrated the ability to break down DMP under the effect of the expression of specific genes associated with energy metabolism and ABC transporters in this bacterium. Notably, under DMP exposure, there was a notable rise in the intermediate metabolites pyruvic acid and citrate, indicating that *Arthrobacter* QD15-4 responded to DMP by modulating its metabolic pathways and transporters [142]. The bacterial strains *Burkholderia zhejiangensis* CEIB S4-3 and *Burkholderia cenocepacia* CEIB S5-2 can break down the pesticide methyl parathion (MP) and its byproduct p-nitrophenol (PNP) due to specific genes in their genomes (*mpd* gene and *pnp* gene cluster) [49] as well as those genes involved in sensing environmental changes, responding to stress, and degrading aromatics. qRT-PCR confirmed their importance in defense against MP and PNP toxicity. Genomic data shows CEIB S5-2 has genes for efficient PNP degradation via different pathways, making it a strong candidate for pesticide removal [143]. A transcriptomic study revealed gene expression changes during MP and PNP breakdown, highlighting roles in energy production, transport, metabolism, and stress response. Transporter genes play a key role in facilitating PNP entry and counteracting its toxicity during biodegradation. Overall, these *Burkholderia* strains possess the genetic tools to degrade MP and PNP effectively, with CEIB S5-2 showing promise for pesticide remediation [144].

### Proteomics and metaproteomics: identification of the active microbial communities and their EOC-biodegradation progress

The profile of proteins, enzymes, and peptides that have different levels of expression in a particular circumstance is referred to as the proteome [128]. A thorough and adequate investigation is produced by proteomics, which offers a detailed analysis of the variations in protein composition as well as how they function and interact. Networks of protein–protein interactions, gene expression, and regulation are studied in proteomics research [145–147]. Predicting microbial functional activities has become feasible, due to the rapid advances of metaproteome and metatranscriptomics (Fig. 2). A new outlook for the biodegradation of toxins is opened by the proteomics approach, which allows to identification and characterization of new proteins that participate in a variety of metabolic pathways including stress response, transportation, energy metabolism, or transcription regulation [80]. Nowadays, proteome research is used directly on the microbial community (meta-proteomics) to understand how they function within a specific ecosystem. This can be very helpful in figuring out whether a microorganism has the ability to break down any compound that is present in the sample [148]. Metaproteomics techniques like Mass Spectrometry (MS) and Two-Dimensional Electrophoresis (2-DE) have advanced our understanding of microbial biodegradation pathways by revealing the key catabolic enzymes involved [129]. For proteomic analysis, a biological specimen must first be prepared. Then, proteins must be extracted and separated using SDS-PAGE or 2D-GE. Next, experimental data must be generated, collected, and analyzed using software for gel image analysis, such as PDQuest (BioRad) and ImageMaster 2D/Melanie. Finally, proteins must be identified and characterized using microbial protein identification technology like MALDI-TOF mass spectrometry. Flex Analysis (v. 3.3) and BioTools may be used to visualize spectra and identify tandem-MS (MS/MS) proteins, and the findings could be connected to commercial proteomic search engines like MASCOT to search for the protein-sequence databases listed under the NCBI number [147, 149].

In a groundbreaking study, Pankaj et al. (2016) pioneered a proteomic approach to investigate the resistance of *Bacillus thuringiensis* SG4 to cypermethrin, a pesticide commonly found in agricultural soil. After a five-day incubation period, the researchers conducted a comparative proteomic analysis of the bacterium with and without exposure to cypermethrin. By extracting the differential whole-cell extracellular proteome from the active bacterial isolate SG4 and analyzing it using 2D electrophoresis, they identified the cypermethrin-resistant proteins in *Bacillus thuringiensis* strain SG4. The analysis also showed that cypermethrin-resistant bacteria exhibited decreased activity in certain dehydrogenase enzymes, including those that act on formate, glycerol-3-phosphate, isocitrate kinase, and phosphatase, as well as malate and ketoglutarate semialdehyde. Furthermore, the induction of cypermethrin led to the down-regulation of the translocase subunit Sec A protein in *Bacillus thuringiensis* SG4. Prominent proteins in the resistant bacteria included NAD kinase, ATPase pumps, ATP synthase, and transferases. The majority of the altered proteins were closely linked to various cellular processes, such as stress response, cypermethrin-degrading catabolism, protein synthesis and modification, gene regulation and transcription, energy production, and chemotaxis [150].

To understand how the strain *S. paucimobilis* 20006FA breaks down phenanthrene and accumulates intermediate metabolites, Macchi et al. (2018) analyzed its genome and compared predictions to experimental proteomic analyses. To investigate the strain's reaction to the phenanthrene, the proteome of *S. paucimobilis* 20006FA was analyzed by 2D-GE. The peptides were evaluated using an ultraviolet MALDITOF/TOF. Proteomic analysis revealed several enzymes were related to transforming phenanthrene into TCA intermediates, which were upregulated by phenanthrene. Analyzing the distinct proteins revealed that they consisted of one NahA1f (alpha subunit of naphthalene dioxygenase), catechol dioxygenase, dihydroxybiphenyl dioxygenase, glutathione S transferases, and various enzymes involved in the lower metabolic pathway such as 2-hydroxymuconic semialdehyde hydrolase and 4-oxalocrotonate decarboxylase, among others. The abundance of dioxygenase enzyme-coding genes in the genome suggested a sizable potential for aromatic biodegradation through the salicylate and protocatechuate pathways. Additionally, this strain was able to degrade other PAHs such as anthracene, dibenzothiophene, and fluoranthene. Through genomic analysis, they identified 126 potential genes that encode enzymes involved in all steps of phenanthrene degradation, which may also be involved in the breakdown of other PAHs [151].

Bastida et al. (2016) examined the effects of oil contamination on the potential of bioremediation using compost amendment. They used fatty acid and metaproteomics analysis to investigate the biomass, evolutionary relationships, and physiological responses of the microbial community in polluted semiarid soils. The fatty-acid analysis was performed by using a Trace Ultra Thermo Scientific gas chromatograph, and Mass spectrometry was used for proteome analysis. They revealed *Proteobacteria* dominated at the phylum level among bacteria with the abundance of *Rhizobiales, Sphingomonadales,* and *Caulobacterales* families, while *Ricketsiales, Rhodospirillales,* and *Rhodobacterales* made up no more than 10% of the *Proteobacterial* proteins. In the contaminated soils, there was a lower concentration of *Rhizobiales* proteins. As opposed to the corresponding control microcosms, *Caulobacterales* significantly increased in the oil-spiked treatments. After compost treatment, *Sphingomonadale* abundance increased, and *Actinobacteria* made up to 17% of the identified bacterial proteins of the microbial community in addition to *Proteobacteria*. Oil pollution caused a decline in *Actinobacterial* proteins and regardless of the various treatments, *Burkholderiales* made up the majority of the identified *Proteobacterial* proteins, accounting for about 95%. *Pseudomonadales* and *Enterobacterales* were the sources of up to 84% of the *Proteobacterial* proteins. *Actinomycetales*, which accounted for nearly all of the *Actinobacterial* proteins and increased in abundance in oil-spiked treatments and compost addition, dominated the *Actinobacteria* phylum. The addition of compost resulted in a significant reduction in PAHs and alkane concentrations that was primarily carried out by *Sphingomonadales* and uncultured bacteria, as their catabolic enzymes' abundance such as catechol and cisdihydrodiol dehydrogenases showed [152].

Williams et al. (2010) investigated the microbial community proteome in toluene-amended soil and its microbial inoculation cultures. They used a Proteomics Analyzer MALDI-TOF/TOF mass spectrometer for protein analysis. The two toluene-impacted groups shared multiple identical proteins, including Tuf (elongation factor Tu), glutamine synthetase, amino acid transporters, extracellular solute-binding proteins, outer membrane proteins, and cell surface-associated proteins like arginine deiminase (ArcA) and ornithine carbamoyltransferase (ArcB). The microbial communities in toluene-affected soil and cultures, but not in those affected by glucose, contained: GroEL (chaperonin), TolC (outer membrane protein), CspA (cold-shock protein), ArcA (arginine deiminase), SucC (succinyl-CoA synthetase), OmpF (outer membrane protein F), succinate dehydrogenase, ABC transporters, glutamate synthetase (Gln), extracellular solute-binding proteins, and outer membrane proteins (Omp). This suggests that these proteins may play a role in toluene removal. CspA and ArcA were present in toluene-amended cultures, while SodB and GroEL were found in microbial protein from toluene-amended soil. 16S rRNA gene analysis of the bacterial communities in toluene-added soil revealed a significant degree of dominance, with members of the *Bacillus* species accounting for 80% of the OTUs. Additionally, the toluene enrichment experiment identified ArcA and CspA, which showed a considerable increase in cultures of *Pseudomonas putida* DOT-T1E grown in the presence of toluene [153].

### Metabolomics: identifying the metabolites during the biodegradation of EOCs

Metabolomics describes the response of microbial communities to particular biological factors, abiotic pressures, and their environment at a given moment. Production of a variety of metabolites or metabolomes in contact with natural environment stimulation is an important part of the study of metabolomics [154, 155]. The development of models that can be used for the prediction of microbiological activity in bioremediation strategies has been facilitated by these approaches [79] (Fig. 2). Researchers have conducted studies to investigate the metabolites produced during the biodegradation of pollutants via two main strategies: global untargeted metabolomics and targeted metabolomics. The first approach is preferred when there is no prior information available to identify the metabolites and generates large amounts of data that can be compared between samples. Using this approach, Keum et al., (2008) analyzed the comparative intracellular metabolome of *Sinorhizobium* sp., including fatty acids, polyhydroxyalkanoates, and polar metabolites during phenanthrene degradation. Their study revealed an increase in the fatty acids profile, TCA and glycolysis intermediates, and accumulation of trehalose as the product of the phenanthrene breakdown in the cell. A few amino acids, such as glycine, homoserine, and valine, also exhibited a rise during the metabolism of phenanthrene. While presence of sulfur amino acids and nicotinic acid showed possible oxidative stress conditions during phenanthrene metabolism [21, 156]. The second metabolomics approach is called targeted metabolomics which is used to identify specific metabolites or metabolic pathways from known databases. The workflow for metabolomics involves selecting a biological sample, extracting and purifying metabolites through chromatography, and then analyzing the data using mass spectrometry or NMR spectroscopy to identify metabolites by comparing them with various databases and libraries [79, 157]. HPLC, GC–MS, and NMR spectroscopy were used by Moody et al. to investigate the metabolism of *Mycobacterium vanbaalenii* strain PYR-1, which degrades benzanthracene. It has been demonstrated that benzanthracene breaks down to generate dihydroxylated and methoxylated intermediates, which are then directed into the main carbon pathway [128, 158]. A frequently used pesticide is carbofuran. The internal and extracellular metabolites of the *Chryseobacterium* sp. BSC2-3 strain, which was chosen from a soil sample, was examined using an LC–MS-based metabolomics approach by Park et al. (2022). The BSC2-3 strain demonstrated the ability to convert carbofuran into 3-hydroxycarbofuran externally. Through intracellular metabolite analysis, it was observed that carbofuran primarily impacted the breakdown of aminobenzoate, the synthesis of ubiquinone and terpenoid-quinone, as well as the metabolism of arginine and proline. Furthermore, the strain was found to produce compounds that induce disease resistance and regulate plant growth. Moreover, the study identified the genes responsible for producing indole-3-acetic acid, a potent auxin [159]. In 2018, Tian et al. used stable isotope-assisted metabolomics (SIAM) to analyze soil contaminated with 13C-labeled fluoranthene, pyrene, and benzoanthracene. They identified metabolites and pathways, detected ring-cleavage products, and found sulfate conjugates of dihydroxy compounds as major metabolites, suggesting that fungi may contribute to the biotransformation of pyrene and benzoanthracene in soil [160].

Fluxomics is a method used to measure and analyze the rates at which metabolic reactions occur and the changes in these rates within a living organism. It involves studying the entire set of metabolic fluxes within a cell, which provides valuable information about various cellular processes. This collection of metabolic fluxes, known as fluxomic, is considered a unique characteristic of the cell. By comparing labeling patterns obtained through appropriate labeling distribution, fluxomics can effectively determine and describe the distribution of metabolic reactions within a cell [20, 21] and aims at capturing the dynamic nature of phenotypes and functional interactions between the genome and the environment [96, 161]. Being based on metabolite data, which is far less abundant than that from proteins and genes, fluxomics offers numerous benefits over proteomics and genomics [162, 163]. However, to the best of our knowledge and according to our searches, there is no report on using this approach in the case of biodegrading potential in the soil.

### Multiomics analysis to unlocking the mechanistic biodegradation of EOCs

Thermophilic bacteria have shown great potential in remediating various pollutants, including azo dyes, DBG, and imidacloprid. Researchers have employed omics approaches, such as proteomics, metabolomics, and genomics, to gain insights into the biodegradation mechanisms employed by these microorganisms. Zhang et al. (2022) isolated a thermophilic bacterial strain, *Anoxybacillus* sp. PDR2, from soil to address azo dye effluent remediation. Through proteomic and metabolomic analyses, they identified crucial transport mechanisms, such as ABC transporters and two-component systems, involved in the stress response. The bacterium was found to self-synthesize a redox mediator, riboflavin, essential for the biodegradation process. *Anoxybacillus* sp. PDR2 utilized glucose as an energy source, employing the TCA cycle and pyruvate metabolism to generate energy in vivo, transferring NAD to the electron transport chain, and ultimately facilitating degradation. Overexpression of acetoacetate synthase and malate synthase G during biodegradation, as indicated by proteomic data, mediated energy supply [164]. An et al. (2020) investigated a thermophilic microbiota's ability to break down DBG using metagenomic sequencing and qRT-PCR to understand gene actions. Through quantitative metaproteomics, they identified specific DBG-degrading associated proteins, such as NADH ubiquinone reductase and NADH-quinone oxidoreductase subunit, linked to the TCA cycle and glycolysis pathways. These proteins produced reducing equivalents crucial for breaking down DBG. Their analysis also highlighted the role of FAD/NAD (P)-binding protein in the biodegradation process [165]. These studies demonstrate the power of omics approaches in unraveling the biodegradation mechanisms employed by thermophilic bacteria. By identifying key enzymes, transport systems, and metabolic pathways involved in pollutant degradation, researchers can develop more efficient bioremediation strategies and optimize the use of these microorganisms in environmental remediation efforts.

Using a multiomics approach, Gautam and colleagues (2023) explored the imidacloprid biodegradation potential of Agrobacterium sp. InxBP2 through whole genome sequencing. Enzymes like FAD-dependent monooxygenase, amidohydrolase family protein, and ABC transporters were identified, showing similarity to known imidacloprid-degrading enzymes. Proteomics analysis revealed distinct metabolic processes in imidacloprid-treated samples compared to controls, a finding supported by metabolomics (GC–MS analysis) [166].

## Conclusions and future perspectives

To successfully remove persistent pollutants like EOCs through biodegradation or bioremediation in their natural environments, it is crucial to understand and recognize the composition, structure, unrealized potential, and interactions between microbial communities and other inhabitants of the surrounding environment. In order to gain a deeper understanding of microbial populations, high-throughput metagenomics and metatranscriptomics would be preferable to traditional sequencing-based methods. However, these innovative methods and approaches are unable to properly address the range of microbes and pathways involved in bioremediation. This might be addressed by employing metaproteomics and metabolomics approaches to precisely identify the enzymes and metabolites present in contaminated areas. The fluxomics approach can also help determine the most significant and effective compounds produced during bioremediation by evaluating the flow and quantity of metabolites under certain conditions. As technology advances in sequencing, chromatography, and mass spectrometry, it is expected that identifying the biodegradation pathways in microorganisms become easier. Despite the developing advancement of omics approaches that led biological science to the "omics age", revolutionized investigation of microbial diversity and their undiscovered bioremediation abilities, uncovering the mysterious properties of this complicated and puissant network requires more multiapproach research to clarify the potential of microbial consortia. Integrated omics allows for the correlation of genome contents with proteins, enzymes, and final metabolites produced during bioremediation in microorganisms making it a standard method for analyzing microbial consortia. This insight enables metaomics to discover all biotechnologically relevant features within a natural consortium. Although omics approaches have significantly advanced our understanding of microbial communities in bioremediation, there are still limitations and potential risks that need to be carefully considered and addressed for successful field-scale implementation. Although omics approaches give extensive insights into microbial communities, there are still gaps in our knowledge of their complex interactions and dynamics in contaminated locations. On the other hand, the analysis and interpretation of huge data sets generated by omics techniques can be complex, demanding specialized bioinformatics tools and knowledge. So, it might be challenging to anticipate unexpected ecological impacts when manipulating microbial populations using omics-guided bioremediation. Furthermore, bioremediation-based techniques may not always result in total pollutant removal, and leftover contaminants might still pose environmental and health concerns. Another gap is overcoming barriers to scaling up omics-based bioremediation technologies from the lab to the field. Soil heterogeneity, fluctuating environmental conditions, and complex pollutant mixes may restrict the direct implementation of lab-based omics findings. Furthermore, it is necessary to integrate omics data with other monitoring and modeling approaches in order to gain a broader view of bioremediation systems. Combining omics data with geochemical data and engineering concepts can result in better-informed soil remediation decisions. Creating numerical models and simulating omics data using sophisticated algorithms to improve the prediction of contamination reduction and microbial metabolism in polluted environments, as well as creating meaningful databases from the massive amount of omics data generated, in order to facilitate knowledge extraction and application in bioremediation may solve some of these limitations.

## Data Availability

The datasets generated during and/or analysed during the current study are available from the corresponding author on reasonable request.

## Abbreviations

TNT: 2,4,6-Trinitrotoluene
2-ADNT: 2-Amino-4,6-Dinitrotoluene
4MBC: 3-(4-Methylbenzylidene) camphor
4-ADNT: 4-Amino-2,6-Dinitrotoluene
AOPs: Advanced oxidation processes
AlkB: Akane monooxygenase
ArcA: Arginine deiminase
ARISA: Automated ribosomal intergenic spacer analysis
BPA: Bisphenol A
CAL: Candida antarctica
CRL: Candida rugosa
CBZ: Carbamazepine
carE: Carboxylesterase gene
CNS: Central nervous system
CP: Chlorpyrifos
COPD: Chronic obstructive pulmonary disease
Csp: Cold-shock protein
DEHP: Di(2-ethylhexyl) phthalate
DDT: Dichlorodiphenyltrichloroethane
DEPs: Differentially expressed proteins
DMs: Differently metabolites
DMP: Dimethyl phthalate
DBG: Direct black G
ECs: Emerging contaminants
EOCs: Emerging organic contaminants
EDCs: Endocrine-disrupting compounds
EHMC: Ethylhexyl methoxycinnamate
Gln: Glutamate synthetase
GOX: Glyphosate oxidase
HCH: Hexachlorocyclohexane
LiP: Lignin peroxidase
MnP: Manganese peroxidase
MS: Mass spectrometry
MALDI-TOF: Matrix-assisted laser desorption ionization time of flight
MBRs: Membrane bioreactors
MP: Methyl parathion
OC: Octadecene
OCPs: Organochlorine pesticides
OP: Organophosphate
OTs: Organotins
ArcB: Ornithine carbamoyltransferase
OPP: Ortho-phenylphenol
Omp: Outer membrane proteins
PacBio: Pacific biosciences
PFCs: Perfluorochemicals
PFOS: Perfluorooctanesulfonate
PFOA: Perfluorooctanoic acid
PCPs: Personal care products
PhACs: Pharmaceuticals
PAEs: Phthalate acid esters
PNP: P-nitrophenol
BPAPC: Poly (bisphenol-A carbonate)
PLA: Poly (lactic acid)
PCL: Poly(ε-caprolactone)
PBDEs: Polybrominated diphenyl ethers
PBS: Polybutylene succinate
PBSA: Polybutylene succinate-co-adipate
PCAs: Polychlorinated alkanes
PCBs: Polychlorinated biphenyls
PCBs: Polychlorinated biphenyls
PCNs: Polychlorinated naphthalenes
PAH: Polycyclic aromatic hydrocarbon
PDMSs: Polydimethylsiloxanes
QACs: Quaternary ammonium compounds
SDS-PAGE: Sodium dodecyl sulfate polyacrylamide gel electrophoresis
SIAM: Stable isotope-assisted metabolomics
SEs: Steroid estrogens
SodB: Superoxide dismutase
TCA: Tricarboxylic acid cycle
TCC: Triclocarban
TCS: Triclosan
2-DE: Two-dimensional electrophoresis

## References

[1] Gogoi A, et al. Occurrence and fate of emerging contaminants in water environment: a review. Groundw Sustain Dev. 2018;6:169–80. 10.1016/j.gsd.2017.12.009. 10.1016/j.gsd.2017.12.009

[2] Wang F, et al. Emerging contaminants: a one health perspective. The Innovation. 2024. 10.1016/j.xinn.2024.100612. 38756954 10.1016/j.xinn.2024.100612 PMC11096751

[3] García J, et al. A review of emerging organic contaminants (EOCs), antibiotic resistant bacteria (ARB), and antibiotic resistance genes (ARGs) in the environment: Increasing removal with wetlands and reducing environmental impacts. Biores Technol. 2020;307: 123228. 10.1016/j.biortech.2020.123228. 10.1016/j.biortech.2020.123228 32247686

[4] Pal A, et al. Emerging contaminants of public health significance as water quality indicator compounds in the urban water cycle. Environ Int. 2014;71:46–62. 10.1016/j.envint.2014.05.025. 24972248 10.1016/j.envint.2014.05.025

[5] Janek G. The uncertainty of biodegradation rate constants of emerging organic compounds in soil and groundwater – a compilation of literature values for 82 substances. Water Res. 2017. 10.1016/j.watres.2017.09.017. 10.1016/j.watres.2017.09.017 28938146

[6] Gaur VK, et al. Integrating advanced techniques and machine learning for landfill leachate treatment: addressing limitations and environmental concerns. Environ Pollut. 2024. 10.1016/j.envpol.2024.124134. 38734050 10.1016/j.envpol.2024.124134

[7] Horta A, et al. Potential of integrated field spectroscopy and spatial analysis for enhanced assessment of soil contamination: a prospective review. Geoderma. 2015;241–242:180–209. 10.1016/j.geoderma.2014.11.024. 10.1016/j.geoderma.2014.11.024

[8] Samal K, Bandyopadhyay R, Dash RR. Biological treatment of contaminants of emerging concern in wastewater: a review. J Hazard Toxic Radioact Waste. 2022. 10.1061/(ASCE)HZ.2153-5515.0000685. 10.1061/(ASCE)HZ.2153-5515.0000685

[9] Vasilachi IC, et al. Occurrence and fate of emerging pollutants in water environment and options for their removal. Water. 2021;13:181. 10.3390/w13020181. 10.3390/w13020181

[10] Ahmed MB, et al. Progress in the biological and chemical treatment technologies for emerging contaminant removal from wastewater: a critical review. J Hazard Mater. 2017;323:274–98. 10.1016/j.jhazmat.2016.04.045. 27143286 10.1016/j.jhazmat.2016.04.045

[11] Morsi R, et al. Laccases and peroxidases: the smart, greener and futuristic biocatalytic tools to mitigate recalcitrant emerging pollutants. Sci Total Environ. 2020;714: 136572. 10.1016/j.scitotenv.2020.136572. 31986384 10.1016/j.scitotenv.2020.136572

[12] Azin E, Jenab K, Moghimi H. Biodegradation of crude oil by a halotolerant and biosurfactant producing strain of *Mucor* circinelloides in different microcosm conditions. Int J Environ Anal Chem. 2022;102(18):7199–208. 10.1080/03067319.2020.1828385. 10.1080/03067319.2020.1828385

[13] Kumar V, et al. Application of omics technologies for microbial community structure and function analysis in contaminated environment. In: Kumar V, et al., editors. Wastewater treatment. Amsterdam: Elsevier; 2021. p. 1–40.

[14] Ghorbannezhad H, Moghimi H, Dastgheib SMM. Evaluation of pyrene and tetracosane degradation by mixed-cultures of fungi and bacteria. J Hazard Mater. 2021;416: 126202. 10.1016/j.jhazmat.2021.126202. 34492965 10.1016/j.jhazmat.2021.126202

[15] Putt AD, RafieHazen S a A ATC. Large-data omics approaches in modern remediation. J Environ Engin. 2022. 10.1061/(ASCE)EE.1943-7870.0002042. 10.1061/(ASCE)EE.1943-7870.0002042

[16] Rodríguez A, et al. Omics approaches to pesticide biodegradation. Curr Microbiol. 2020;77:545–63. 10.1007/s00284-020-01916-5. 32078006 10.1007/s00284-020-01916-5

[17] Bala S, et al. Recent strategies for bioremediation of emerging pollutants: a review for a green and sustainable environment. Toxics. 2022;10(8):484. 10.3390/toxics10080484. 36006163 10.3390/toxics10080484 PMC9413587

[18] Chaurasia P, Jasuja ND, Kumar S. Bioremediation assessment in industrial wastewater treatment: the omics approach. In: Kumar V, Thakur IS, editors. Omics insights in environmental bioremediation. Singapore: Springer Nature Singapore; 2022. p. 455–85.

[19] Sharma P, et al. Omics approaches in bioremediation of environmental contaminants: an integrated approach for environmental safety and sustainability. Environ Res. 2022;211: 113102. 10.1016/j.envres.2022.113102. 35300964 10.1016/j.envres.2022.113102

[20] Sharma P, et al. Microbial fingerprinting techniques and their role in the remediation of environmental pollution. Clean Chem Engin. 2022;2: 100026. 10.1016/j.clce.2022.100026. 10.1016/j.clce.2022.100026

[21] Malla MA, et al. Understanding and designing the strategies for the microbe-mediated remediation of environmental contaminants using omics approaches. Front Microbiol. 2018;9:1132. 10.3389/fmicb.2018.01132. 29915565 10.3389/fmicb.2018.01132 PMC5994547

[22] Kumar V, Thakur IS. Omics insights in environmental bioremediation. Singapore: Springer; 2022.

[23] Gupta A, et al. Rhizospheric remediation of organic pollutants from the soil; a green and sustainable technology for soil clean up. In: Gupta A, et al., editors. Abatement of environmental pollutants. Amsterdam: Elsevier; 2020. p. 263–86.

[24] Saravanan A, et al. Microorganism-mediated bioremediation of dyes from contaminated soil: mechanisms, recent advances, and future perspectives. Food Chem Toxicol. 2024. 10.1016/j.fct.2024.114491. 38325634 10.1016/j.fct.2024.114491

[25] Kaur I, et al. Plants exert beneficial influence on soil microbiome in a HCH contaminated soil revealing advantage of microbe-assisted plant-based HCH remediation of a dumpsite. Chemosphere. 2021;280: 130690. 10.1016/j.chemosphere.2021.130690. 34162081 10.1016/j.chemosphere.2021.130690

[26] Predikaka T, et al. A full-scale bioremediation study of diesel fuel-contaminated soil: the effect of plant species and soil amendments. Int J Environ Sci Technol. 2024;21(4):4319–30. 10.1007/s13762-023-05304-x. 10.1007/s13762-023-05304-x

[27] Tijani JO, et al. Pharmaceuticals, endocrine disruptors, personal care products, nanomaterials and perfluorinated pollutants: a review. Environ Chem Lett. 2016;14(1):27–49. 10.1007/s10311-015-0537-z. 10.1007/s10311-015-0537-z

[28] Mukhopadhyay A, Duttagupta S, Mukherjee A. Emerging organic contaminants in global community drinking water sources and supply: a review of occurrence, processes and remediation. J Environ Chem Eng. 2022;10(3): 107560. 10.1016/j.jece.2022.107560. 10.1016/j.jece.2022.107560

[29] Lei M, et al. Overview of emerging contaminants and associated human health effects. Biomed Res Int. 2015. 10.1155/2015/404796. 26713315 10.1155/2015/404796 PMC4680045

[30] Rani L, et al. An extensive review on the consequences of chemical pesticides on human health and environment. J Clean Prod. 2021;283: 124657. 10.1016/j.jclepro.2020.124657. 10.1016/j.jclepro.2020.124657

[31] Rykowska I, Wasiak W. Research trends on emerging environment pollutants - a review. Open Chem. 2016. 10.1515/chem-2015-0151. 10.1515/chem-2015-0151

[32] Gavrilescu M, et al. Emerging pollutants in the environment: present and future challenges in biomonitoring, ecological risks and bioremediation. N Biotechnol. 2015;32(1):147–56. 10.1016/j.nbt.2014.01.001. 24462777 10.1016/j.nbt.2014.01.001

[33] Quintella CM, Mata AMT, Lima LCP. Overview of bioremediation with technology assessment and emphasis on fungal bioremediation of oil contaminated soils. J Environ Manag. 2019;241:156–66. 10.1016/j.jenvman.2019.04.019. 10.1016/j.jenvman.2019.04.019 30999265

[34] Mousavi SA, Khodadoost F. Effects of detergents on natural ecosystems and wastewater treatment processes: a review. Environ Sci Poll Res. 2019;26(26):26439–48. 10.1007/s11356-019-05802-x. 10.1007/s11356-019-05802-x 31352596

[35] Wang H, et al. Ecotoxicological effects, environmental fate and risks of pharmaceutical and personal care products in the water environment: a review. Sci Total Environ. 2021;788: 147819. 10.1016/j.scitotenv.2021.147819. 34029823 10.1016/j.scitotenv.2021.147819

[36] Song X, et al. Environmental risk assessment of the emerging EDCs contaminants from rural soil and aqueous sources: analytical and modelling approaches. Chemosphere. 2018;198:546–55. 10.1016/j.chemosphere.2018.01.060. 29433099 10.1016/j.chemosphere.2018.01.060

[37] Li N, et al. Effects of endocrine disrupting chemicals in host health: three-way interactions between environmental exposure, host phenotypic responses, and gut microbiota. Environ Pollut. 2021;271:116387. 10.1016/j.envpol.2020.116387. 33401209 10.1016/j.envpol.2020.116387

[38] Werkneh AA, et al. Removal of endocrine disrupters from the contaminated environment: public health concerns, treatment strategies and future perspectives—a review. Heliyon. 2022. 10.1016/j.heliyon.2022.e09206. 35464705 10.1016/j.heliyon.2022.e09206 PMC9026580

[39] Yilmaz B, et al. Endocrine disrupting chemicals: exposure, effects on human health, mechanism of action, models for testing and strategies for prevention. Rev Endocr Metab Disord. 2020;21(1):127–47. 10.1007/s11154-019-09521-z. 31792807 10.1007/s11154-019-09521-z

[40] Sharma I. Bioremediation techniques for polluted environment: concept, advantages, limitations, and prospects. In: Sharma I, editor. Trace metals in the environment-new approaches and recent advances. London: IntechOpen; 2020. p. 221–36.

[41] Sharma P, et al. Recent advancements in microbial-assisted remediation strategies for toxic contaminants. Clean Chem Engin. 2022;2: 100020. 10.1016/j.clce.2022.100020. 10.1016/j.clce.2022.100020

[42] Sales da Silva IG, et al. Soil bioremediation: overview of technologies and trends. Energies. 2020;13(18):4664. 10.3390/en13184664. 10.3390/en13184664

[43] Kumar V, Shahi SK, Singh S, et al. Bioremediation: an eco-sustainable approach for restoration of contaminated sites. In: Singh J, et al., editors. Microbial bioprospecting for sustainable development. Singapore: Springer Singapore; 2018. p. 115–36.

[44] Liu S-H, et al. Bioremediation mechanisms of combined pollution of PAHs and heavy metals by bacteria and fungi: a mini review. Biores Technol. 2017;224:25–33. 10.1016/j.biortech.2016.11.095. 10.1016/j.biortech.2016.11.095 27916498

[45] Giyahchi M, Moghimi H. Aerobic biodegradation of untreated polyester–polyether urethanes by newly isolated yeast strains *Exophilia* sp. NS-7 and *Rhodotorula* sp. NS-12. Scientific reports. 2023;13(1):5016. 10.1038/s41598-023-31639-z. 36977741 10.1038/s41598-023-31639-z PMC10050204

[46] Dave S, Das J. Chapter 13 - Role of microbial enzymes for biodegradation and bioremediation of environmental pollutants: challenges and future prospects. In: Saxena G, Kumar V, Shah MP, editors. Bioremediation for environmental sustainability. Amsterdam: Elsevier; 2021. p. 325–46.

[47] Haripriyan U, et al. Bioremediation of organic pollutants: a mini review on current and critical strategies for wastewater treatment. Arch Microbiol. 2022;204(5):286. 10.1007/s00203-022-02907-9. 35478273 10.1007/s00203-022-02907-9

[48] Tripathi V, et al. Assessing the half-life and degradation kinetics of aliphatic and aromatic hydrocarbons by bacteria isolated from crude oil contaminated soil. Chemosphere. 2023;337: 139264. 10.1016/j.chemosphere.2023.139264. 37348617 10.1016/j.chemosphere.2023.139264

[49] Amaro Bittencourt G, et al. Emerging contaminants bioremediation by enzyme and nanozyme-based processes—a review. iScience. 2023;26(6): 106785. 10.1016/j.isci.2023.106785. 37250780 10.1016/j.isci.2023.106785 PMC10209495

[50] Karigar CS, Rao SS. Role of microbial enzymes in the bioremediation of pollutants: a review. Enzym Res. 2011;2011: 805187. 10.4061/2011/805187. 10.4061/2011/805187 PMC3168789 21912739

[51] Jenab K, Moghimi H, Azin E. Crude oil and pyrene degradation by halotolerant fungi *Embellisia* sp. KJ59 and *Alternaria* sp. KJ66 isolated from saline soils. Int J Environ Anal Chem. 2023;103(17):5453–64. 10.1080/03067319.2021.1939023. 10.1080/03067319.2021.1939023

[52] Moghimi H, Heidary Tabar R, Hamedi J. Assessing the biodegradation of polycyclic aromatic hydrocarbons and laccase production by new fungus *Trematophoma* sp. UTMC 5003. World J Microbiol Biotechnol. 2017;33:1–10. 10.1007/s11274-017-2304-8. 28585171 10.1007/s11274-017-2304-8

[53] Park JW, Park BK, Kim JE. Remediation of soil contaminated with 2,4-dichlorophenol by treatment of minced shepherd’s purse roots. Arch Environ Contam Toxicol. 2006;50(2):191–5. 10.1007/s00244-004-0119-8. 16392021 10.1007/s00244-004-0119-8

[54] González-González RB, et al. Bio-removal of emerging pollutants by advanced bioremediation techniques. Environ Res. 2022. 10.1016/j.envres.2022.113936. 35932833 10.1016/j.envres.2022.113936

[55] Azin E, Moghimi H, Heidarytabar R. Petroleum degradation, biosurfactant and laccase production by *Fusarium neocosmosporiellum* RH-10: a microcosm study. Soil Sediment Contamination Int J. 2018;27(4):329–42. 10.1080/15320383.2018.1473334. 10.1080/15320383.2018.1473334

[56] Xu M, et al. Critical role of monooxygenase in biodegradation of 2, 4, 6-Trinitrotoluene by *Buttiauxella* sp. S19–1. Molecules. 2023;28(4):1969. 10.3390/molecules28041969. 36838956 10.3390/molecules28041969 PMC9958683

[57] Cao Z, et al. Construction of microbial consortia for microbial degradation of complex compounds. Front Bioeng Biotechnol. 2022;10:1051233. 10.3389/fbioe.2022.1051233. 36561050 10.3389/fbioe.2022.1051233 PMC9763274

[58] Ali SS, et al. Plastic wastes biodegradation: mechanisms, challenges and future prospects. Sci Total Environ. 2021;780: 146590. 10.1016/j.scitotenv.2021.146590. 34030345 10.1016/j.scitotenv.2021.146590

[59] Saravanan A, et al. A review on catalytic-enzyme degradation of toxic environmental pollutants: microbial enzymes. J Hazard Mater. 2021;419: 126451. 10.1016/j.jhazmat.2021.126451. 34174628 10.1016/j.jhazmat.2021.126451

[60] Ghorbannezhad H, Moghimi H, Dastgheib SMM. Biodegradation of high molecular weight hydrocarbons under saline condition by halotolerant *Bacillus* subtilis and its mixed cultures with *Pseudomonas* species. Sci Rep. 2022;12(1):13227. 10.1038/s41598-022-17001-9. 35918482 10.1038/s41598-022-17001-9 PMC9345985

[61] Robles-Morales DL, et al. Design and performance evaluation of a fungi-bacteria consortium to biodegrade organic matter at high concentration on synthetic slaughterhouse wastewater. Water Air Soil Pollut. 2021;232(6):223. 10.1007/s11270-021-05177-1. 10.1007/s11270-021-05177-1

[62] Cheng Z, et al. Improved biodegradation potential of chlorobenzene by a mixed fungal-bacterial consortium. Int Biodeterioration Biodegradation. 2017;123:276–85. 10.1016/j.ibiod.2017.07.008. 10.1016/j.ibiod.2017.07.008

[63] Kurade MB, et al. Monitoring the gradual biodegradation of dyes in a simulated textile effluent and development of a novel triple layered fixed bed reactor using a bacterium-yeast consortium. Chem Eng J. 2017;307:1026–36. 10.1016/j.cej.2016.09.028. 10.1016/j.cej.2016.09.028

[64] Kumar M, et al. Antibiotics bioremediation: perspectives on its ecotoxicity and resistance. Environ Int. 2019;124:448–61. 10.1016/j.envint.2018.12.065. 30684803 10.1016/j.envint.2018.12.065

[65] Cunningham CJ, et al. Potential risks of antibiotic resistant bacteria and genes in bioremediation of petroleum hydrocarbon contaminated soils. Environ Sci Process Impact. 2020;22(5):1110–24. 10.1039/c9em00606k. 10.1039/c9em00606k 32236187

[66] Kumar V, Shahi S, Singh S. Bioremediation: an eco-sustainable approach for restoration of contaminated sites. Microbial Bioprospecting Sust Dev. 2018. 10.1007/978-981-13-0053-0_6. 10.1007/978-981-13-0053-0_6

[67] Parveen S, Asgher M, Bilal M. Lignin peroxidase-based cross-linked enzyme aggregates (LiP-CLEAs) as robust biocatalytic materials for mitigation of textile dyes-contaminated aqueous solution. Environ Technol Innov. 2021;21: 101226. 10.1016/j.eti.2020.101226. 10.1016/j.eti.2020.101226

[68] Sharma B, Dangi AK, Shukla P. Contemporary enzyme based technologies for bioremediation: a review. J Environ Manag. 2018;210:10–22. 10.1016/j.jenvman.2017.12.075. 10.1016/j.jenvman.2017.12.075 29329004

[69] Barrios-Estrada C, et al. Potentialities of active membranes with immobilized laccase for bisphenol A degradation. Int J Biol Macromol. 2018;108:837–44. 10.1016/j.ijbiomac.2017.10.177. 29101049 10.1016/j.ijbiomac.2017.10.177

[70] Dodor DE, Hwang H-M, Ekunwe SIN. Oxidation of anthracene and benzo[a]pyrene by immobilized laccase from *Trametes* versicolor. Enzym Microb Technol. 2004;35(2):210–7. 10.1016/j.enzmictec.2004.04.007. 10.1016/j.enzmictec.2004.04.007

[71] Singh SK, et al. Chapter 6—microbial enzymes and their exploitation in remediation of environmental contaminants. In: Kumar A, et al., editors. Microbe mediated remediation of environmental contaminants. Sawston: Woodhead Publishing; 2021. p. 59–71.

[72] Scott C, et al. A free-enzyme catalyst for the bioremediation of environmental atrazine contamination. J Environ Manag. 2010;91(10):2075–8. 10.1016/j.jenvman.2010.05.007. 10.1016/j.jenvman.2010.05.007 20570036

[73] Scott C, et al. The enzymatic basis for pesticide bioremediation. Indian J Microbiol. 2008;48(1):65–79. 10.1007/s12088-008-0007-4. 23100701 10.1007/s12088-008-0007-4 PMC3450202

[74] Zainith S, et al. 9—microbial ligninolytic enzymes and their role in bioremediation. In: Chowdhary P, et al., editors. Microorganisms for sustainable environment and health. Amsterdam: Elsevier; 2020. p. 179–203.

[75] Bhandari S, et al. Microbial enzymes used in bioremediation. J Chem. 2021;2021:8849512. 10.1155/2021/8849512. 10.1155/2021/8849512

[76] Kumar A, et al. Microbial lipolytic enzymes—promising energy-efficient biocatalysts in bioremediation. Energy. 2020;192: 116674. 10.1016/j.energy.2019.116674. 10.1016/j.energy.2019.116674

[77] Narayanan M, Ali SS, El-Sheekh M. A comprehensive review on the potential of microbial enzymes in multipollutant bioremediation: mechanisms, challenges, and future prospects. J Environ Manag. 2023;334: 117532. 10.1016/j.jenvman.2023.117532. 10.1016/j.jenvman.2023.117532 36801803

[78] Biswas R, Sarkar A, et al. ‘Omics’ tools in soil microbiology: the state of the art. In: Adhya TK, et al., editors. Advances in soil microbiology: recent trends and future prospects: volume 1: soil-microbe interaction. Singapore: Springer Singapore; 2018. p. 35–64.

[79] Chandran H, Meena M, Sharma K. Microbial biodiversity and bioremediation assessment through omics approaches. Front Environ Chem. 2020. 10.3389/fenvc.2020.570326. 10.3389/fenvc.2020.570326

[80] Aziz A, et al. Chapter 25—Genomics in understanding bioremediation of inorganic pollutants. In: Hasanuzzaman M, Prasad MNV, editors., et al., Handbook of bioremediation. Cambridge: Academic press; 2021. p. 397–410.

[81] Villa-Rodríguez ED, Díaz-Rodríguez AM, de Los Santos Villalobos S. (2024) *Omics approaches for detecting action modes of microbial inoculants.* In: Villa-Rodríguez ED, Díaz-Rodríguez AM, de Los Santos Villalobos S* (Eds.) New Insights, Trends, and Challenges in the Development and Applications of Microbial Inoculants in Agriculture*. Elsevier: Amsterdam. Pp. 69–86

[82] Vannier N, et al. Genome-resolved metatranscriptomics reveals conserved root colonization determinants in a synthetic microbiota. Nat Commun. 2023;14(1):8274. 10.1038/s41467-023-43688-z. 38092730 10.1038/s41467-023-43688-z PMC10719396

[83] Sandrini M, et al. Abiotic stress and belowground microbiome: the potential of omics approaches. Int J Mol Sci. 2022;23(3):1091. 10.3390/ijms23031091. 35163015 10.3390/ijms23031091 PMC8835006

[84] Kaur H, et al. Integrating omics technologies for a comprehensive understanding of the microbiome and its impact on cattle production. Biology. 2023;12(9):1200. 10.3390/biology12091200. 37759599 10.3390/biology12091200 PMC10525894

[85] Jain A, et al. Omics approaches in understanding the benefits of plant-microbe interactions. Front Microbiol. 2024;15:1391059. 10.3389/fmicb.2024.1391059. 38860224 10.3389/fmicb.2024.1391059 PMC11163067

[86] Bhimani P, et al. Unveiling the green dialogue: advancements in omics technologies for deciphering plant–microbe interactions in soil. Discover Plant. 2024;1(1):4. 10.1007/s44372-024-00004-3. 10.1007/s44372-024-00004-3

[87] González-Plaza JJ, et al. Advances in experimental and computational methodologies for the study of microbial-surface interactions at different omics levels. Front Microbiol. 2022;13:1006946. 10.3389/fmicb.2022.1006946. 36519168 10.3389/fmicb.2022.1006946 PMC9744117

[88] Riyaz M, Raj K. Emerging microbial identification technologies in the era of OMICS and genome editing. In: Riyaz M, Raj K, editors. Role of microbes in sustainable development: human health and diseases. Singapore: Springer; 2023.

[89] Janiszewska D, et al. “Omic” approaches to bacteria and antibiotic resistance identification. Int J Mol Sci. 2022;23(17):9601. 10.3390/ijms23179601. 36077000 10.3390/ijms23179601 PMC9455953

[90] Pande V, et al. Bioremediation: an emerging effective approach towards environment restoration. Environ Sustain. 2020;3:91–103. 10.1007/s42398-020-00099-w. 10.1007/s42398-020-00099-w

[91] Thomas T, Gilbert J, Meyer F. Metagenomics-a guide from sampling to data analysis. Microb Inf Exp. 2012;2:1–12. 10.1186/2042-5783-2-3. 10.1186/2042-5783-2-3 PMC3351745 22587947

[92] Zhang L, et al. Advances in metagenomics and its application in environmental microorganisms. Front Microbiol. 2021;12: 766364. 10.3389/fmicb.2021.766364. 34975791 10.3389/fmicb.2021.766364 PMC8719654

[93] Lapidus AL, Korobeynikov AI. Metagenomic data assembly–the way of decoding unknown microorganisms. Front Microbiol. 2021;12: 613791. 10.3389/fmicb.2021.613791. 33833738 10.3389/fmicb.2021.613791 PMC8021871

[94] Ottoni J, et al. Metagenomic approaches applied to bioremediation of xenobiotics. In: Kumar V, Bilal M, Ferreira LF, Iqbal HMN, editors., et al., Genomics approach to bioremediation: principles, tools, and emerging technologies. Hoboken: Wiley; 2023. p. 125–42.

[95] Ottoni JR, et al. Functional metagenomics of oil-impacted mangrove sediments reveals high abundance of hydrolases of biotechnological interest. World J Microbiol Biotechnol. 2017. 10.1007/s11274-017-2307-5. 28593475 10.1007/s11274-017-2307-5

[96] Datta S, et al. Metagenomic applications in microbial diversity, bioremediation, pollution monitoring, enzyme and drug discovery. a review. Environ Chem Lett. 2020;18:1229–41. 10.1007/s10311-020-01010-z. 10.1007/s10311-020-01010-z

[97] Shyam, K., et al., 2023. *Omics technologies in environmental microbiology and microbial ecology insightful aapplications in bioremediation research*. In: V Kumar, M Bilal, LFR Ferreira, HMN Iqbal (Eds.) *Genomics approach to bioremediation principles, tools, and emerging technologies*. Pp.433–454.

[98] Lagier JC, et al. Culturing the human microbiota and culturomics. Nat Rev Microbiol. 2018;16:540–50. 10.1038/s41579-018-0041-0. 29937540 10.1038/s41579-018-0041-0

[99] Tripathi M, et al. Metagenomic approach towards bioprospection of novel biomolecule(s) and environmental bioremediation. Ann Res Rev Biol. 2018;22:1–12. 10.9734/ARRB/2018/38385. 10.9734/ARRB/2018/38385

[100] Nowrotek M, et al. Culturomics and metagenomics: in understanding of environmental resistome. Front Environ Sci Engin. 2019;13(3):40. 10.1007/s11783-019-1121-8. 10.1007/s11783-019-1121-8

[101] Desai C, Pathak H, Madamwar D. Advances in molecular and “-omics” technologies to gauge microbial communities and bioremediation at xenobiotic/anthropogen contaminated sites. Biores Technol. 2010;101(6):1558–69. 10.1016/j.biortech.2009.10.080. 10.1016/j.biortech.2009.10.080 19962886

[102] Mishra S, et al. Recent advanced technologies for the characterization of xenobiotic-degrading microorganisms and microbial communities. Front Bioengin Biotechnol. 2021. 10.3389/fbioe.2021.632059. 10.3389/fbioe.2021.632059 PMC7902726 33644024

[103] Jadeja NB, Kapley A. Designing knowledge-based bioremediation strategies using metagenomics. In: Jadeja NB, Kapley A, editors. Metagenomic data analysis. Singapore: Springer; 2023. p. 195–208. 10.1007/978-1-0716-3072-3_9 37258863

[104] Garrido-Sanz D, et al. Metagenomic analysis of a biphenyl-degrading soil bacterial consortium reveals the metabolic roles of specific populations. Front Microbiol. 2018. 10.3389/fmicb.2018.00232. 29497412 10.3389/fmicb.2018.00232 PMC5818466

[105] Ibrahim SAEM, El-Bialy HA, Gomaa OM. Biodegradation of COVID19 antibiotic; azithromycin and its impact on soil microbial community in the presence of phenolic waste and with temperature variation. World J Microbiol Biotechnol. 2023;39(6):154. 10.1007/s11274-023-03591-7. 37037954 10.1007/s11274-023-03591-7 PMC10085964

[106] Bhardwaj P, et al. Atrazine bioremediation and its influence on soil microbial diversity by metagenomics analysis. Indian J Microbiol. 2020;60(3):388–91. 10.1007/s12088-020-00877-4. 32647398 10.1007/s12088-020-00877-4 PMC7329956

[107] Zhao S, Wang J. Biodegradation of atrazine and nicosulfuron by *Streptomyces nigra* LM01: performance, degradative pathway, and possible genes involved. J Hazard Mater. 2024. 10.1016/j.jhazmat.2024.134336. 38640665 10.1016/j.jhazmat.2024.134336

[108] Duarte M, et al. Functional soil metagenomics: elucidation of polycyclic aromatic hydrocarbon degradation potential following 12 years of in situ bioremediation. Environ Microbiol. 2017;19:2992–3011. 10.1111/1462-2920.13756. 28401633 10.1111/1462-2920.13756

[109] Zang H, et al. Carboxylesterase, a de-esterification enzyme, catalyzes the degradation of chlorimuron-ethyl in *Rhodococcus**erythropolis* D310–1. J Hazard Mater. 2020;387:121684. 10.1016/j.jhazmat.2019.121684. 31784128 10.1016/j.jhazmat.2019.121684

[110] Bhende RS, Bombaywala S, Dafale NA. Unleashing potential of *Pseudomonas**aeruginosa* RNC3 and Stenotrophomonas maltophilia RNC7 for chlorpyrifos biodegradation by genome analysis and kinetic studies. J Hazard Mater. 2024;461: 132668. 10.1016/j.jhazmat.2023.132668. 37793258 10.1016/j.jhazmat.2023.132668

[111] Cao X, et al. Biodegradation properties and mechanism of triketone herbicide mesotrione via newly isolated bacterium Klebsiella pasteurii CM-1. Int Biodeterior Biodegradation. 2024;187: 105727. 10.1016/j.ibiod.2023.105727. 10.1016/j.ibiod.2023.105727

[112] Sun S, et al. Characterization of a novel amidohydrolase with promiscuous esterase activity from a soil metagenomic library and its application in degradation of amide herbicides. Environ Sci Poll Res. 2024. 10.1007/s11356-024-32362-6. 10.1007/s11356-024-32362-6 PMC10948491 38383926

[113] Cota-Ruiz K, et al. A comparative metagenomic and spectroscopic analysis of soils from an international point of entry between the US and Mexico. Environ Int. 2019;123:558–66. 10.1016/j.envint.2018.12.055. 30622080 10.1016/j.envint.2018.12.055

[114] Regar RK, et al. Comparative microbiome analysis of two different long-term pesticide contaminated soils revealed the anthropogenic influence on functional potential of microbial communities. Sci Total Environ. 2019;681:413–23. 10.1016/j.scitotenv.2019.05.090. 31108361 10.1016/j.scitotenv.2019.05.090

[115] Wang W, et al. Complete genome sequence of the cyprodinil-degrading bacterium Acinetobacter johnsonii LXL_C1. Microb Pathog. 2019;127:246–9. 10.1016/j.micpath.2018.11.016. 30496837 10.1016/j.micpath.2018.11.016

[116] Gangola S, et al. Differential analysis of pesticides biodegradation in soil using conventional and high-throughput technology. bioRxiv. 2021. 10.1101/2021.06.01.446544. 10.1101/2021.06.01.446544

[117] Redfern LK, et al. A new framework for approaching precision bioremediation of PAH contaminated soils. J Hazard Mater. 2019;2019(378):120859. 10.1016/j.jhazmat.2019.120859. 10.1016/j.jhazmat.2019.120859 PMC6833951 31327574

[118] McLain NK, Gomez MY, Gachomo EW. Acetaminophen levels found in recycled wastewater alter soil microbial community structure and functional diversit. Microbial Ecol. 2023;85(4):1448–62. 10.1007/s00248-022-02022-8. 10.1007/s00248-022-02022-8 PMC10167187 35507048

[119] Smułek W, et al. Bacteria involved in biodegradation of creosote PAH - a case study of long-term contaminated industrial area. Ecotoxicol Environ Saf. 2020;187:109843. 10.1016/j.ecoenv.2019.109843. 31678701 10.1016/j.ecoenv.2019.109843

[120] Bouhajja E, et al. Identification of novel toluene monooxygenase genes in a hydrocarbon-polluted sediment using sequence- and function-based screening of metagenomic libraries. Appl Microbiol Biotechnol. 2017;101(2):797–808. 10.1007/s00253-016-7934-5. 27785541 10.1007/s00253-016-7934-5

[121] Azam S, et al. Genome organization and adaptive potential of archetypal organophosphate degrading *Sphingobium**fuliginis* ATCC 27551. Genom Biol Evol. 2019;11(9):2557–62. 10.1093/gbe/evz189. 10.1093/gbe/evz189 PMC6934885 31504476

[122] Benedek T, et al. Nocardioides *carbamazepini* sp. nov., an ibuprofen degrader isolated from a biofilm bacterial community enriched on carbamazepine. Syst Appl Microbiol. 2022;45(4):126339. 10.1016/j.syapm.2022.126339. 35714383 10.1016/j.syapm.2022.126339

[123] Kumar R, et al. Landfill microbiome harbour plastic degrading genes: a metagenomic study of solid waste dumping site of Gujarat, India. Sci Total Environ. 2021;779:146184. 10.1016/j.scitotenv.2021.146184. 33752005 10.1016/j.scitotenv.2021.146184

[124] Zhu F, et al. Metagenomic analysis exploring microbial assemblages and functional genes potentially involved in di (2-ethylhexyl) phthalate degradation in soil. Sci Total Environ. 2020;715: 137037. 10.1016/j.scitotenv.2020.137037. 32041058 10.1016/j.scitotenv.2020.137037

[125] Cai Y, et al. Metagenomic analysis of soil microbial community under PFOA and PFOS stress. Environ Res. 2020;188:109838. 10.1016/j.envres.2020.109838. 32798955 10.1016/j.envres.2020.109838

[126] Thelusmond J-R, Strathmann TJ, Cupples AM. Carbamazepine, triclocarban and triclosan biodegradation and the phylotypes and functional genes associated with xenobiotic degradation in four agricultural soils. Sci Total Environ. 2019;657:1138–49. 10.1016/j.scitotenv.2018.12.145. 30677881 10.1016/j.scitotenv.2018.12.145

[127] Bokade P, et al. Bacterial remediation of pesticide polluted soils: exploring the feasibility of site restoration. J Hazard Mater. 2023;441:129906. 10.1016/j.jhazmat.2022.129906. 36088882 10.1016/j.jhazmat.2022.129906

[128] Mohapatra B, et al. Microbial metabolism of aromatic pollutants: High-throughput OMICS and metabolic engineering for efficient bioremediation. In: Mohapatra B, et al., editors. Current developments in biotechnology and bioengineering. Amsterdam: Elsevier; 2022. p. 151–99.

[129] Shyam K, et al. Omics technologies in environmental microbiology and microbial ecology. In: Shyam K, et al., editors. Genomics approach to bioremediation. Hoboken p: Wiley; 2023. p. 433–54.

[130] Rodríguez A, et al. Omics approaches to pesticide biodegradation. Curr Microbiol. 2020;77(4):545–63. 10.1007/s00284-020-01916-5. 32078006 10.1007/s00284-020-01916-5

[131] Sharma PK, et al. Comparative metatranscriptome analysis revealed broad response of microbial communities in two soil types, agriculture versus organic soil. J Genet Eng Biotechnol. 2019;17(1):6. 10.1186/s43141-019-0006-3. 31659568 10.1186/s43141-019-0006-3 PMC6821142

[132] Cheng Y, et al. Global transcriptomic analysis of Rhodococcus erythropolis D310–1 in responding to chlorimuron-ethyl. Ecotoxicol Environ Safe. 2018;157:111–20. 10.1016/j.ecoenv.2018.03.074. 10.1016/j.ecoenv.2018.03.074 29614448

[133] Brzeszcz J, et al. Hydrocarbon removal by two differently developed microbial inoculants and comparing their actions with biostimulation treatment. Molecules. 2020;25(3):661. 10.3390/molecules25030661. 32033085 10.3390/molecules25030661 PMC7036810

[134] Sharma R, Sharma PK. Metatranscriptome sequencing and analysis of agriculture soil provided significant insights about the microbial community structure and function. Ecological Genet Genom. 2018;6:9–15. 10.1016/j.egg.2017.10.001. 10.1016/j.egg.2017.10.001

[135] Li Y, et al. Changes in microbial community structure and co-metabolism during the domestication of ofloxacin-degrading bacteria. Environ Sci Eur. 2022;34(1):1–16. 10.1186/s12302-022-00691-3. 10.1186/s12302-022-00691-3

[136] Russell JN, et al. Metagenomic and metatranscriptomic analysis reveals enrichment for xenobiotic-degrading bacterial specialists and xenobiotic-degrading genes in a Canadian prairie two-cell biobed system. Environ Microbiol Rep. 2021;13(5):720–7. 10.1111/1758-2229.12990. 34236147 10.1111/1758-2229.12990

[137] Singh DP, et al. Metatranscriptome analysis deciphers multifunctional genes and enzymes linked with the degradation of aromatic compounds and pesticides in the wheat rhizosphere. Front Microbiol. 2018;9:1331. 10.3389/fmicb.2018.01331. 30034370 10.3389/fmicb.2018.01331 PMC6043799

[138] Tartaglia M, et al. Exploring an enhanced rhizospheric phenomenon for pluricontaminated soil remediation: Insights from tripartite metatranscriptome analyses. J Hazard Mater. 2022;428:128246. 10.1016/j.jhazmat.2022.128246. 35030484 10.1016/j.jhazmat.2022.128246

[139] de Menezes A, Clipson N, Doyle E. Comparative metatranscriptomics reveals widespread community responses during phenanthrene degradation in soil. Environmental Microbiology. 2012;14(9):2577–88. 10.1111/j.1462-2920.2012.02781.x. 22625871 10.1111/j.1462-2920.2012.02781.x

[140] Yergeau E, et al. Microbial expression profiles in the rhizosphere of willows depend on soil contamination. The ISME J. 2014;8(2):344–58. 10.1038/ismej.2013.163. 24067257 10.1038/ismej.2013.163 PMC3906822

[141] Pagé AP, Yergeau É, Greer CW. Salix purpurea stimulates the expression of specific bacterial xenobiotic degradation genes in a soil contaminated with hydrocarbons. PloS One. 2015;10(7):e0132062–e0132062. 10.1371/journal.pone.0132062. 26161539 10.1371/journal.pone.0132062 PMC4498887

[142] Wang C, et al. Response of Arthrobacter QD 15–4 to dimethyl phthalate by regulating energy metabolism and ABC transporters. Ecotoxicol Environ Saf. 2019;174:146–52. 10.1016/j.ecoenv.2019.02.078. 30825737 10.1016/j.ecoenv.2019.02.078

[143] Ortiz-Hernández ML, et al. Transcriptomic analysis of Burkholderia cenocepacia CEIB S5–2 during methyl parathion degradation. Environ Sci Pollut Res. 2021;28(31):42414–31. 10.1007/s11356-021-13647-6. 10.1007/s11356-021-13647-6 33813711

[144] Castrejón-Godínez ML, et al. Transcriptional analysis reveals the metabolic state of *Burkholderia**zhejiangensis* CEIB S4–3 during methyl parathion degradation. PeerJ. 2019;7:e6822–e6822. 31086743 10.7717/peerj.6822 PMC6486813

[145] Arora PK, Srivastava A, Singh VP. Bacterial degradation of nitrophenols and their derivatives. J Hazard Mater. 2014;266:42–59. 10.1016/j.jhazmat.2013.12.011. 24374564 10.1016/j.jhazmat.2013.12.011

[146] Wasinger VC, Corthals GL. Proteomic tools for biomedicine. J Chromatogr B Analyt Technol Biomed Life Sci. 2002;771(1–2):33–48. 10.1016/s1570-0232(02)00125-3. 12015991 10.1016/s1570-0232(02)00125-3

[147] Gangola S, et al. Biotechnological tools to elucidate the mechanism of pesticide degradation in the environment. Chemosphere. 2022;296:133916. 10.1016/j.chemosphere.2022.133916. 35149016 10.1016/j.chemosphere.2022.133916

[148] Wang D-Z, et al. Environmental microbial community proteomics: status, challenges and perspectives. Int J Mol Sci. 2016;17(8):1275. 10.3390/ijms17081275. 27527164 10.3390/ijms17081275 PMC5000673

[149] Pandey A, et al. Omics technology to study bioremediation and respective enzymes. In: Pandey A, et al., editors. Smart bioremediation technologies. Amsterdam p: Elsevier; 2019. p. 23–43.

[150] Pankaj, et al. Differential expression and characterization of cypermethrin-degrading potential proteins in Bacillus thuringiensis strain, SG4. 3 Biotech. 2016;6:1–13. 10.1007/s13205-016-0541-4. 10.1007/s13205-016-0541-4 PMC5071268 28330297

[151] Macchi M, et al. Insights into the genome and proteome of Sphingomonas paucimobilis strain 20006FA involved in the regulation of polycyclic aromatic hydrocarbon degradation. World J Microbiol Biotechnol. 2018;34:1–14. 10.1007/s11274-017-2391-6. 10.1007/s11274-017-2391-6 29214360

[152] Bastida F, et al. The ecological and physiological responses of the microbial community from a semiarid soil to hydrocarbon contamination and its bioremediation using compost amendment. J Proteomics. 2016;135:162–9. 10.1016/j.jprot.2015.07.023. 26225916 10.1016/j.jprot.2015.07.023

[153] Williams MA, Taylor EB, Mula HP. Metaproteomic characterization of a soil microbial community following carbon amendment. Soil Biol Biochem. 2010;42(7):1148–56. 10.1016/j.soilbio.2010.03.021. 10.1016/j.soilbio.2010.03.021

[154] Miller MG. Environmental metabolomics: a SWOT analysis (strengths, weaknesses, opportunities, and threats). J Proteome Res. 2007;6(2):540–5. 10.1021/pr060623x. 17269710 10.1021/pr060623x

[155] Kumar V, Thakur IS, Shah MP. Bioremediation approaches for treatment of pulp and paper industry wastewater: recent advances and challenges. Microbial Bioremed Biodegradation. 2020. 10.1007/978-981-15-1812-6_1. 10.1007/978-981-15-1812-6_1

[156] Keum YS, et al. Comparative metabolomic analysis of *Sinorhizobium* sp. C4 during the degradation of phenanthrene. Appl Microbiol Biotechnol. 2008;80:863–72. 10.1007/s00253-008-1581-4. 18668240 10.1007/s00253-008-1581-4 PMC7419452

[157] Alonso A, Marsal S, Julià A. Analytical methods in untargeted metabolomics: state of the art in 2015. Front Bioengin biotechnol. 2015;3:23. 10.3389/fbioe.2015.00023 PMC4350445 25798438

[158] Moody JD, Freeman JP, Cerniglia CE. Degradation of benz [a] anthracene by *Mycobacterium* vanbaalenii strain PYR-1. Biodegradation. 2005;16:513–26. 10.1007/s10532-004-7217-1. 15865344 10.1007/s10532-004-7217-1

[159] Park H, et al. Screening of carbofuran-degrading bacteria *Chryseobacterium* sp. BSC2–3 and unveiling the change in metabolome during carbofuran degradation. Metabolites. 2022;12(3):219. 10.3390/metabo12030219. 35323662 10.3390/metabo12030219 PMC8950912

[160] Tian Z, et al. Tracing the biotransformation of polycyclic aromatic hydrocarbons in contaminated soil using stable isotope-assisted metabolomics. Environ Sci Technol Lett. 2018;5(2):103–9. 10.1021/acs.estlett.7b00554. 31572742 10.1021/acs.estlett.7b00554 PMC6767928

[161] Jhariya U, et al. Understanding the role of genetic and protein networking involved in microbial bioremediation. Bioremed Environ Pollut Emerg Trend Strateg. 2022. 10.1007/978-3-030-86169-8. 10.1007/978-3-030-86169-8

[162] Charu, et al. Recent trends in metagenomic approaches in environmental cleanup, in omics insights in environmental bioremediation. Singapore: Springer; 2022. p. 605–24.

[163] Cascante M, Marin S. Metabolomics and fluxomics approaches. Essay Biochem. 2008;45:67–82. 10.1042/bse0450067. 10.1042/bse0450067 18793124

[164] Zhang S, et al. Molecular response of *Anoxybacillus* sp. PDR2 under azo dye stress: an integrated analysis of proteomics and metabolomics. J Hazard Mater. 2022;438:129500. 10.1016/j.jhazmat.2022.129500. 35792431 10.1016/j.jhazmat.2022.129500

[165] An X, et al. Integrated metagenomic and metaproteomic analyses reveal potential degradation mechanism of azo dye-direct black G by thermophilic microflora. Ecotoxicol Environ Saf. 2020;196: 110557. 10.1016/j.ecoenv.2020.110557. 32259760 10.1016/j.ecoenv.2020.110557

[166] Gautam P, Pandey AK, Dubey SK. Multi-omics approach reveals elevated potential of bacteria for biodegradation of imidacloprid. Environ Res. 2023;221: 115271. 10.1016/j.envres.2023.115271. 36640933 10.1016/j.envres.2023.115271

